# Exploration of Quasi‐Direct Band Edge in a Multilayer Ferroelectric Semiconductor for Applications in Van Der Waals Stacked Heterojunction Solar Cell and Photocatalytic Devices

**DOI:** 10.1002/advs.202514717

**Published:** 2025-10-08

**Authors:** Anna Milatul Ummah, Yen‐Chang Su, Yu‐Hung Peng, You‐Xun Xu, Ching‐Hwa Ho

**Affiliations:** ^1^ Graduate Institute of Applied Science and Technology National Taiwan University of Science and Technology Taipei 106 Taiwan; ^2^ Taiwan Consortium of Emergent Crystalline Materials (TCECM) National Science and Technology Council Taipei 106 Taiwan

**Keywords:** heterojunction device, luminescence, optical property, photocatalysis, quasi‐direct bandgap, solar cell

## Abstract

AgBiP_2_Se_6_ is a ferroelectric semiconductor with a Curie temperature above 300 K which also possesses a variety of functional capabilities. In this work, it is demonstrated that despite its intrinsically indirect bandgap, multilayer (ML) AgBiP_2_Se_6_ exhibits unexpectedly strong photoluminescence, attributed to its quasi‐direct band structure. The energy difference between the indirect and direct transitions is relatively small (≈0.075 eV), as confirmed by both theoretical calculations and experimental observations. Temperature‐dependent optical measurements, corroborated by electronic band structure analysis, reveal the coexistence of indirect ($E_{1}^{\mathrm{ind}}$) and direct ($E_{2}^{d}$) bandgaps, with phonon‐assisted processes playing a significant role in the material's optoelectronic behaviors. The $E_{1}^{\mathrm{ind}}$ and $E_{2}^{d}$ transitions at 300 K are determined to be 1.46 and 1.535 eV, respectively. The indirect transition $E_{1}^{\mathrm{ind}}$ is confirmed by transmittance (T) measurement, while the direct transition $E_{2}^{d}$ is simultaneously detected through micro‐photoluminescence (µPL) and micro‐thermoreflectance (µTR) measurements. A stacked p‐Ga_0.5_In_0.5_Se/n‐AgBiP_2_Se_6_ heterojunction solar cell is successfully fabricated, achieving a photoelectric conversion efficiency (PCE) up to ≈0.583%. Furthermore, AgBiP_2_Se_6_ is demonstrated as a promising photocatalyst, exhibiting a high degradation efficiency to organic dyes. ML‐AgBiP_2_Se_6_ exhibits a quasi‐direct bandgap and ferroelectric behavior at room temperature, making it a strong candidate for next‐generation electronic, optoelectronic, and environment‐protection functions.

## Graphical abstract

Multilayered AgBiP_2_Se_6_ has been systematically characterized to be a quasi‐direct semiconductor using theoretical calculations and optical experiments. It possesses an indirect bandgap of 1.46 eV and a direct gap of 1.535 eV for simultaneously absorbing the NIR to visible‐light photons. In combination with a p‐type Ga_0.5_In_0.5_Se, a high efficiency p‐Ga_0.5_In_0.5_Se/n‐AgBiP_2_Se_6_ stacked solar cell is thus fabricated and demonstrated in this work.

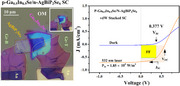

## 1 Introduction

Bandgap engineering and accommodation including compositional bandgap tuning, thickness‐dependent bandgap modulation, indirect, direct, and quasi‐direct bandgap etc. are crucial for designing novel materials in various functional applications. A quasi‐direct semiconductor is characterized by the conduction band minimum (CBM) and valence band maximum (VBM) are located at slightly different momentum values in k‐spaces but still permit for relatively efficient radiative recombination. These materials exhibit hybrid electronic properties that encompass features of both direct and indirect bandgap semiconductors. They typically have an indirect bandgap in bulk material but exhibit direct‐like characteristics under certain conditions (such as strain, constituent, and quantum confinement, etc.).^[^ ^1^, ^2^, ^3^ ^]^ While direct semiconductors (e.g., GaAs) are more efficient for light emission,^[^ ^4^, ^5^, ^6^ ^]^ quasi‐direct (semi‐direct) semiconductors have unique advantage that makes them valuable in specific applications, particularly in optoelectronics and photovoltaics,^[^ ^7^, ^8^, ^9^ ^]^ also in photocatalyst^[^ ^10^ ^]^ due to the coexistence of both direct and indirect bandgaps for photons’ absorption. These materials reveal relatively lower light emission efficiency as compared to direct semiconductors, but are compensated by advantages in thermal management, integration, and tunability of optical and optoelectronic properties.^[^ ^11^, ^12^ ^]^

AgBiP_2_Se_6_, belonging to the group of layered bimetallic thiophosphate ABP_2_X_6_ (A═Ag, Cu, Mn, Li; B═Bi, Cr, In, Al, Fe, V; X═S, Se), has attracted attention for its specific physical properties and potential in advanced materials applications. These materials are crystallized in a rhombohedral structure and have been isolated by weak van der Waals (vdW) forces, enabling mechanical exfoliation down to nano‐scale, like other well‐known 2D materials such as MoS_2_ or graphene.^[^ ^13^, ^14^ ^]^ AgBiP_2_Se_6_ exhibits an intrinsic ferroelectricity driven by its lack of inversion symmetry, especially in its monolayer form where the polarization is easy to control by electric field.^[^ ^15^, ^16^ ^]^ This ferroelectric (FE) behavior, combined with its semiconducting nature and relatively narrow bandgap (≈1.3–1.5 eV), makes AgBiP_2_Se_6_ as a promising material for use in non‐volatile memory devices,^[^ ^17^ ^]^ 2D ferroelectric field‐effect transistors,^[^ ^18^, ^19^ ^]^ and photodetectors.^[^ ^19^, ^20^ ^]^ In addition to the ferroelectricity, AgBiP_2_Se_6_ exhibits strong optical anisotropy, nonlinear optical properties, and orientation‐dependent electrical transport due to its layered anisotropic crystal structure. Previous studies have revealed its potential capability in photovoltaic effects,^[^ ^21^ ^]^ strain‐tunable optoelectronics,^[^ ^18^ ^],^ and ionic transport^[^ ^19^ ^]^ where the movement of Ag^+^ ions may contribute to retain switching behavior for ferroelectricity.

Beyond the above‐mentioned functions, AgBiP_2_Se_6_ might interestingly possess quasi‐direct semiconductor characteristic. Ju et al. reported that the energy difference between direct and indirect band gaps for ferroelectric (FE) and paraelectric (PE) AgBiP_2_Se_6_ monolayers is around 80 and 50 meV, respectively, based on the HSE06 functional calculations.^[^ ^22^ ^]^ Zhang et al. also reported that the 2D‐AgBiP_2_Se_6_ is an indirect bandgap semiconductor with high absorption coefficient and which can achieve indirect to direct transformation by applying uniaxial and biaxial strains.^[^ ^23^ ^]^ Additionally, AgBiP_2_Se_6_ had also exhibited a weak and broadened photoluminescence (PL) in a 20‐nm‐thickness sample.^[^ ^19^ ^]^ So far, most of the research works on optical and electrical properties of AgBiP_2_Se_6_ had only been focused on the theoretical methods.^[^ ^24^ ^]^ There is rare study on the experimental result of emission structure and band edge transition of the multilayer AgBiP_2_Se_6_ that had been characterized to date. Nevertheless, the quasi‐direct semiconductor must be attractive for optoelectronic applications because they combine the strong light absorption of direct bandgap materials with the relatively long carrier lifetimes typically from indirect bandgap semiconductors. It makes multilayered AgBiP_2_Se_6_ more promising for application in photovoltaic devices, photodetectors, and photocatalysis, where efficiently absorbing photons and reducing recombination are both achieved.

In this work, optical and electrical properties of AgBiP_2_Se_6_ have been explored in detail and analyzed using both theoretical calculations and experimental studies. We conduct band structure calculations using Quantum ESPRESSO to identify the indirect and direct band edges of AgBiP_2_Se_6_. We also systematically perform micro‐photoluminescence (µPL) experiment of multilayer (ML) AgBiP_2_Se_6_ of different thicknesses to investigate direct‐gap light‐emission behavior and also compare it to micro‐thermoreflectance (µTR) and transmittance measurements. The results show a quasi‐direct band edge constructed in ML‐AgBiP_2_Se_6_ with small energy difference existed in between the direct and indirect bandgaps. Moreover, the application in stacked heterojunction and photocatalyst have been successfully demonstrated. We conducted a p‐Ga_0.5_In_0.5_Se/n‐AgBiP_2_Se_6_ stacked heterojunction, performing the diode characteristic and achieving high solar‐cell performance of the device structure. We also demonstrate AgBiP_2_Se_6_ as a good candidate for photocatalyst, proven by high degradation efficiency with methylene blue (MB) as the tested pollutant target in water enviroment.

## 2 Results and Discussion

### 2.1 Structural and Raman Characterizations

**Figure 1:**
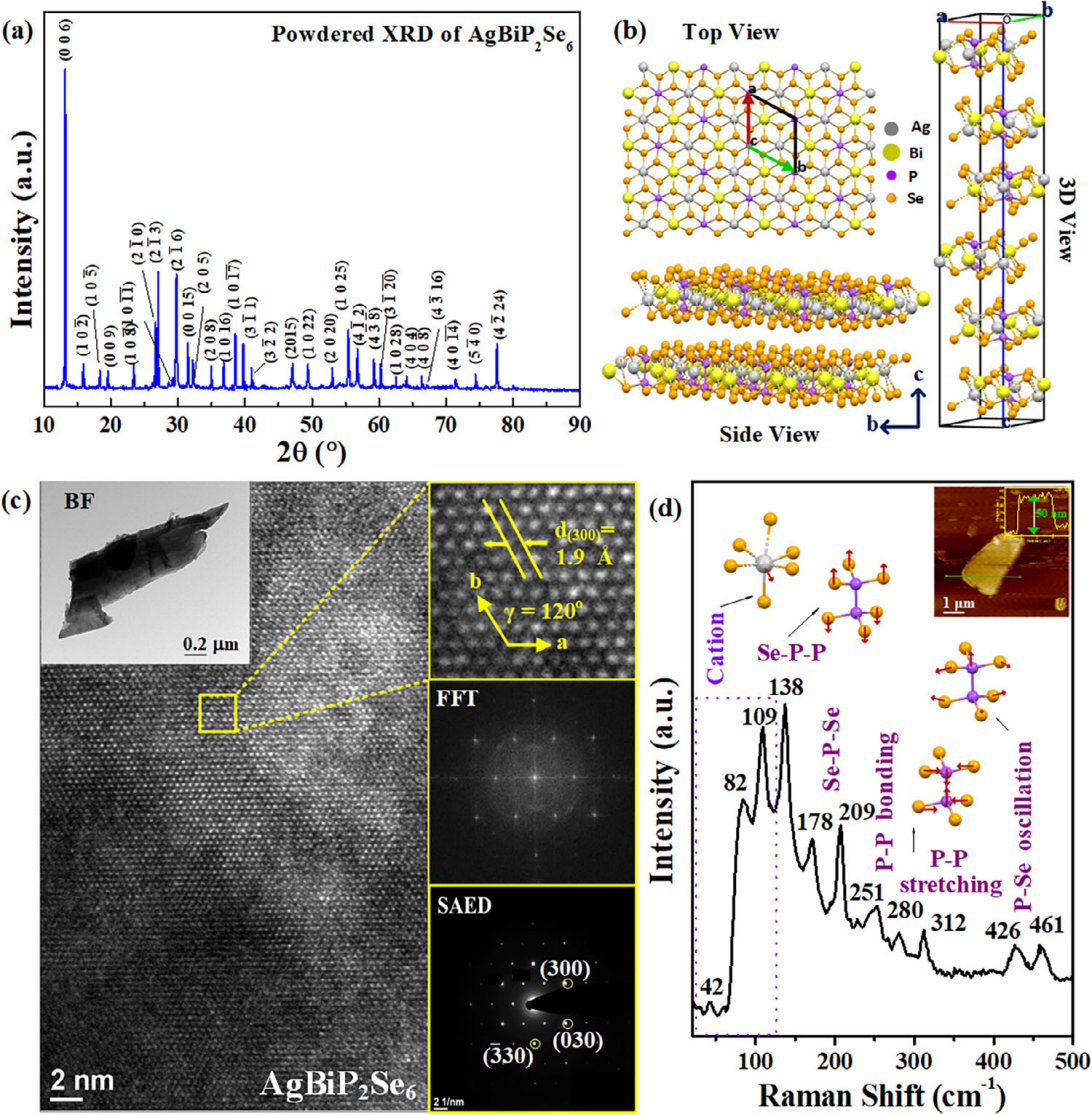
Structural properties of AgBiP_2_Se_6_. a) Experimental XRD pattern of powdered AgBiP_2_Se_6_. b) Crystal structure of AgBiP_2_Se_6_ viewed along the layer plane (top view), the a axis (side view), and the unit cell of AgBiP_2_Se_6_ (3D view), with Ag atoms in silver spheres, Bi atoms in yellow spheres, P atoms in purple spheres, and Se atoms in orange spheres are shown. c) High‐resolution transmission electron microscope (HRTEM) image, bright field (BF) mode nanoflake, fast fourier transform (FFT) result, selection‐area electron diffraction (SAED) pattern of AgBiP_2_Se_6_ nanoflakes. d) Micro‐Raman spectra of multilayered AgBiP_2_Se_6_ of thickness ≈50 nm. The inset shows atomic‐force microscopy (AFM) result.

The high quality AgBiP_2_Se_6_ single crystals were successfully grown using CVT (Chemical Vapor Transport) method with the temperature control and setting shown in Figure S1(Supporting Information). **Figure** **1**a shows the powder X‐ray diffraction (XRD) pattern to determine the crystallographic phase of as‐grown AgBiP_2_Se_6_, and the stoichiometric content was also confirmed by energy‐dispersive X‐ray spectroscopy (EDS) in Figure S2 (Supporting Information). The obtained XRD pattern in Figure 1a is well‐matched with the AgBiP_2_Se_6_ standard ICDD (PDF‐2 Card #01‐073‐3555) and displays the sharp diffraction peaks belonging to AgBiP_2_Se_6_ phase and without obvious impurity‐phase related peaks. The as‐grown AgBiP_2_Se_6_ crystals reveal high purity and remain ambient stability under air condition. The crystal structure of AgBiP_2_Se_6_ belongs to a rhombohedral space group R $\bar{3}$ at room temperature (RT) with the calculated lattice parameters of a = b = 6.59 Å and c = 39.66 Å, which shows a good agreement with previous reports.^[^ ^15^, ^20^, ^25^ ^]^ Figure 1b displays the atomic structure of AgBiP_2_Se_6_, adopting a lamellar FePSe_3_ type structure. The cations and P–P coupling occupy the octahedral holes in each layer, which are specified by the selenium frameworks in AgBiP_2_Se_6_. An ordered configuration of interlayer Ag^+^ and Bi^3+^ cation centers bridged the [P_2_Se_6_]^4−^ anion clusters constructed in the layers. Due to the elongation of three Ag‐Se bonds (can be seen in top, side or 3D view in Figure 1b), AgBiP_2_Se_6_ exhibits distortion of both Ag^+^ and Bi^3+^ ions from the octahedral framework of selenide (Se) atoms at RT, generating anti‐ferroelectric distortion configuration. Notably in a one‐unit cell, AgBiP_2_Se_6_ consists of a sixfold superstructure (6 layers) along the c‐axis which are held together by van der Waals (vdW) interactions. It is also noted that the monolayer has a thickness of ≈0.7 nm, defined as the sum of the atomic layer thickness (d_1L_) and the interlayer gap (d_gap_) between two adjacent layers.

The high‐resolution transmission electron microscope (HRTEM) image in Figure 1c was conducted to further investigate atomic‐scale structure and crystallinity of AgBiP_2_Se_6_ derived from layered AgBiP_2_Se_6_ nanoribbon flakes as shown in the inset (the left‐upper part). The lattice spacing value of d_(300)_ is 1.9 Å with γ is equal to 120° as shown in the magnification image from a square area (see the right‐upper part). The result clearly exhibits atomic dot structure to show high crystallinity of AgBiP_2_Se_6_. The fast‐Fourier‐transform (FFT) pattern obtained from the HRTEM result is consistent with the selected‐area electron diffraction (SAED) result. In accordance with the HRTEM lattice spacing and published data,^[^ ^19^, ^21^ ^]^ the SAED pattern displays a dotted pattern corresponding to the indexed planes of (300), (030) and $(\bar{3}30).$ Due to rhombohedral AgBiP_2_Se_6_ lacks an inversion center as compared to the hexagonal structure, the Bi and Ag ions are not symmetrically equivalent, then R $\bar{3}$ (centrosymmetric) is too restrictive, resulting in symmetry‐forbidden conditions [such as (100), (200), etc. that can be displayed in hexagonal system] and some symmetry like (300), (330) will have strong spots revealed in the SAED pattern in Figure 1c.

The intrinsic vibrational properties and crystalline quality of the as‐grown ML‐AgBiP_2_Se_6_ are examined by Raman spectroscopy, as presented in Figure 1d. The inset image presents the atomic force microscopy (AFM) image of an exfoliated ML‐AgBiP_2_Se_6_ on Si/SiO_2_ substrate used during the characterization in this work. It presents the thickness of sample is about 50 nm, corresponds to ≈71 layers or ≈12 unit cells. The µRaman spectra of ML‐AgBiP_2_Se_6_ at RT shows obvious 11 Raman modes: A_g_ – 42 cm^−1^, A_g_ – 82 cm^−1^, E_g_ ‐109 cm^−1^, E_g_ – 138 A_g_ – cm^−1^, A_g_ – 178 cm^−1^, A_g_ – 209 cm^−1^, A_g_ – 251 cm^−1^, E_g_ – 280 cm^−1^, A_g_ – 312 cm^−1^, A_g_ – 426 cm^−1^ and A_g_ – 461 cm^−1^ with different attributions for each mode, revealing a series of well‐resolved vibrational modes between 40 and 500 cm^−1^. This result is in a good consistent with prior research findings^[^ ^19^, ^20^ ^]^ and similar to the MPX_3_ (metal phosphorous trichalcogenides) characteristics.^[^ ^26^, ^27^ ^]^ Raman spectroscopy provides critical insights into the lattice dynamics and bonding characteristics of layered materials. In this case, the analysis uncovers several vibrational signatures attributed to the constituent atomic interactions within the AgBiP_2_Se_6_ lattice. The low‐frequency peaks below 120 cm^−1^ are assigned to be lattice vibrations involving the cationic sublattice (Ag^+^ and Bi^3+^) and coupled modes associated with Se─P─P units. These low‐frequency features, especially ≈ 42 cm^−1^, are often indicative of interlayer shear and breathing modes, commonly observed in van der Waals‐layered materials.^[^ ^28^ ^]^ The prominent peaks between 120 and 400 cm^−1^ correspond to internal vibrations of the Se─P─Se and P─P─P structural motifs, consistent with reported vibrational assignments in related metal phosphochalcogenides.^[^ ^29^ ^]^ These modes reflect the stretching and bending vibrations involving P atoms in various bonding environments, a key signature of the P‐P unit embedded within the lattice. These peaks confirm the preservation of phosphorus dimerization, which plays a crucial role in defining the electronic and vibrational properties of the crystal.^[^ ^27^ ^]^ The higher‐frequency modes above 400 cm^−1^ are associated with P–Se bond oscillations, which arise from the terminal Se atoms bonded to phosphorus. These modes typically represent localized vibrational modes and are sensitive to subtle structural perturbations, such as strain or doping. In addition, the angle‐dependent polarized µRaman from 0° to 180° and the temperature‐dependent µRaman results in ML‐AgBiP_2_Se_6_ are presented in Figures S3 and S4 (Supporting Information), respectively. The angular dependence of polarized µRaman spectra at 300 K is carried out and the results are shown in Figure S3a (Supporting Information), presenting an anisotropy behavior in rhombohedral AgBiP_2_Se_6_. Figure S3b (Supporting Information) shows the polarized µRaman spectra measured under two polarization configurations: electric field perpendicular to the b‐axis [E⊥b (90° – Z(XY)$\bar{Z}$)], and electric field parallel to the b‐axis [E//b (0° – Z(XX)$\bar{Z}$) or (180° – Z(X $\bar{X}$)$\bar{Z}$], which can be observed in Figure S3a (Supporting Information) in detail for each configuration angle. The intensity changing of each Raman mode is also demonstrated in Figure S3c (Supporting Information) using contour plot. Furthermore, the polar plot derived from the µRaman polarization is shown in Figure S3d (Supporting Information) for each Raman mode and the fitting parameters are listed in Table S1 (Supporting Information). The circle dots indicate experimental data points, while the solid lines represent least‐squares fits using a dichroic model describing the variation of Raman intensity with polarization angle expressed as:

(1) $$I_{\theta }=I_{0}+I_{P}.\mathrm{co}s^{2}\left(\theta -\theta_{m}\right)$$

where I_0_ denotes the minimum Raman intensity as the change of polarization angle in Figure S3a (Supporting Information) observed when the polarization is parallel to the b‐axis (at 90°, E//b), I_P_ is the maximum intensity of Raman peak, and θ_m_ indicates the angular offset from the direction of maximum intensity, which aligns perpendicular to the b‐axis (at 0°, E⊥b). The Raman modes below 140 cm^−1^ and all the A_g_ modes show the perfect “8” shape polar plot with intensity become fully forbidden in θ = 90°, indicating the anisotropic characteristics of the out‐of‐plane vibration modes in AgBiP_2_Se_6_ similar to the other 2D layer semiconductors of GaTe and GeS.^[^ ^30^, ^31^ ^]^

Figure S4a (Supporting Information) depicts the temperature‐dependent µRaman of ML‐AgBiP_2_Se_6_ measured from 300 K to 10 K. All of the Raman modes shift to the higher wavenumber with temperature decreasing and which also enhanced in intensity, following the general semiconductor behavior. The temperature‐dependent µRaman spectra confirmed that the linear shifting in Raman frequency (Figure S4b, Supporting Information), reflecting bond‐length vibrations, is directly related to the thermal dilation and contraction of the crystal lattice as the temperature varies, consistent with Vegard's law of a crystalline lattice. The Raman shift with temperature can be described by linear equation: ν_i_(T) = ν_i_(0) – m_i_.T cm^−1^ (Figure S4c,d, Supporting Information) with the fitted parameters and attributions listed in Table S2 (Supporting Information), corresponding to lattice expansion in AgBiP_2_Se_6_. The results present the slope with m_i_ of ≈2.99 × 10^−3^ to 10.3 × 10^−3^ cm^−1^⋅K^−1^, being in alignment with prior findings in the other layered MPX_3_.^[^ ^26^, ^27^ ^]^

### 2.2 Indirect Bandgap and Quasi‐Direct Band Nature of AgBiP_2_Se_6_

**Figure 2:**
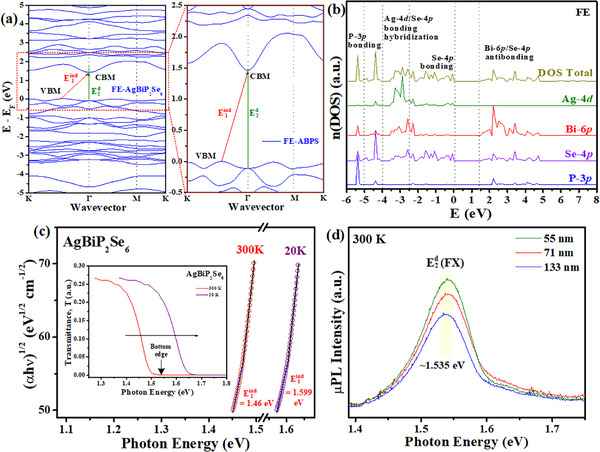
Band structure, DOS, direct and indirect transition of AgBiP_2_Se_6_. a) Electronic band structure of single‐layer ferroelectric (FE) AgBiP_2_Se_6_. b) Normalized partial density of states (PDOS) and total density of states (DOS) in FE‐AgBiP_2_Se_6_. c) The analysis of (αhν)^1/2^ vs. hν spectra at 300 and 20 K for obtaining the indirect bandgap E_g_ (i.e. E_1_^ind^) and averaged phonon energy E_p_ in Equations (2)–(4). The inset shows the transmittance spectra of ML‐AgBiP_2_Se_6_ at 300 and 20 K. d) The micro‐photoluminescence (µPL) spectra of a thin layer FE‐AgBiP_2_Se_6_ (with three different‐thickness parts of 55, 71, and 133 nm) to evaluate direct bandgap (E_2_^d^) transition of the free‐exciton (FX) emission peak at 300 K.

AgBiP_2_Se_6_ exhibits specific quasi‐direct bandgap characteristic, where its electronic band structure is nominally indirect while the direct‐transition energy is very close to the fundamental indirect bandgap. This subtle phenomenon is crucial for understanding its strong photoluminescence (PL) emission despite its indirect band structure. The as‐grown AgBiP_2_Se_6_ layered crystals grown by CVT demonstrate ferroelectric behavior at RT, and the electric‐polarization current‐voltage (*I–V*) experiment of bulk layered AgBiP_2_Se_6_ had been implemented and shown in Figure S5 (Supporting Information). Figure S5 (Supporting Information) depicts the bidirectional *I–V* result of a 76‐µm thick (area 0.1 × 0.2 cm^2^) AgBiP_2_Se_6_ and the applied voltages are between −3 and 3 V on both end electrodes of the van der Waals planes depicted in the inset. The positive and negative voltages induced dipole polarizations of each region and the polarized schemes of the dipoles are also included and indicated in Figure S5 (Supporting Information) for comparison. The *I–V* curves reveal ferroelectric hysteresis behavior for the multilayer AgBiP_2_Se_6_. It reveals a FE (Ferroelectric) performance and which agrees well with the other FE sample,^[^ ^32^ ^]^ and also similar to some of the previous works of the FE materials.^[^ ^33^, ^34^ ^]^ In the present study, we focus on experimental and theoretical characterizations of the layered FE‐AgBiP_2_Se_6_ band structure, and some of the investigated results are demonstrated in **Figure** **2**. The left panel in Figure 2a shows the calculated electronic band structure of single‐layer AgBiP_2_Se_6_ using density functional theory (DFT) in the E‐E_F_ range of −5 to 5 eV, where E = E_F_ (0 eV) marks the energy position of valence band maximum (VBM). The right part in Figure 2a highlights the magnification of the band structure in the E‐E_F_ range of −0.5 to 2.5 eV for AgBiP_2_Se_6_. An indirect bandgap ($E_{1}^{\mathrm{ind}}$) is observed from the VBM at the Γ‐K direction and transits to the conduction band minimum (CBM) that located at Γ point with an indirect transition energy of 1.483 eV. Meanwhile, the direct transition ($E_{2}^{d}$) is observed at the Γ point with a transition energy of 1.57 eV for the direct bandgap. The energy difference of indirect and direct bandgaps is only 0.087 eV (in accordance with previous report^[^ ^22^ ^]^) that renders AgBiP_2_Se_6_ a quasi‐direct bandgap semiconductor resembled with those of the other band structures on 2D MoSi_2_N_4_^[^ ^35^ ^]^ and Si_10_.^[^ ^7^ ^]^ The quasi‐direct band nature of AgBiP_2_Se_6_ with increasing thickness to few‐layer or multilayer forms remains unchanged while their indirect and direct bandgaps are simultaneously reduced with increasing thickness based on theoretical DFT calculations. At this stage, we only calculate the single‐layer FE and PE (Figure 2a; Figure S6a, Supporting Information) band structure for proving the quasi‐direct band structure of the layered AgBiP_2_Se_6_ herein.

Figure 2b shows the calculated total and projected density of states (PDOS) of FE‐AgBiP_2_Se_6_ between −6 and 8 eV close to band edge. The conduction band (CB) state primarily consists of Se 4*p* and Bi 6*p* antibonding states, while the VB portion is dominated by the hybridization of Ag 4*d* and Se 4*p* bonding states. The VBM is mainly dominant by Se 4*p* bonding state. The sharp DOS features near the band edges suggest relatively localized states, consistent with the intrinsic property of van der Waals layered structure and weak interlayer coupling in 2D materials. These orbital‐resolved contributions reflect the hybridization between Se, P orbitals and the delocalization of Bi and Ag states, which is crucial for understanding optoelectronic properties of FE‐AgBiP_2_Se_6_. Additionally, band structure calculations and the PDOS result of the paraelectric (PE) AgBiP_2_Se_6_ can be observed in Figure S6a,b (Supporting Information). The result also shows indirect band edge of PE‐AgBiP_2_Se_6_ with the indirect (i.e. M – K → Γ) and direct (i.e. Γ → Γ) bandgaps are around 1.434 and 1.496 eV, respectively. The DFT band‐structure calculations in Figure 2a and Figure S6a (Supporting Information) confirm the indirect‐gap nature of both FE and PE phases in AgBiP_2_Se_6_ with their detailed calculated data together with previously reported theoretical values of bandgap are listed in Table S3 (Supporting Information) for comparison. It is noted that the bandgap of the PE phase AgBiP_2_Se_6_ is lower than that of the FE phase,^[^ ^36^ ^]^ however, the determination of the crystalline phase in either FE or PE modification depends on the growth temperature of AgBiP_2_Se_6_. FE‐AgBiP_2_Se_6_ is easy to form at a low temperature and which will become a PE phase at even higher temperatures.^[^ ^15^ ^]^ The smaller bandgap of the PE phase (as comparing to the FE phase) is owing to the relaxation of structural distortion and symmetry breaking in the AgBiP_2_Se_6_ lattice. The FE phase usually exhibits stronger localization and asymmetry near band edge as displayed in the comparison of PDOS of the FE and PE phases in Figure S6c (Supporting Information), particularly in the Bi and Se contributions, reflecting the broken inversion symmetry and internal electric polarization. The PE phase in Figure S6c (Supporting Information) maintains centrosymmetric structure with relatively delocalized charge distributions, whereas the FE phase exhibits stronger electronic localization and modified orbital interactions, which directly influence the material's optoelectronic and electrical properties.

Experimental verification of the quasi‐direct band edge of the ML‐AgBiP_2_Se_6_ is also implanted by optical characterization. Transmittance and absorption results are typically used for determining bandgap of semiconductors. As shown in Figure 2c, spectral analysis^[^ ^37^ ^]^ using optical‐absorption coefficient with (𝛼hν)^1/2^ versus hν that had been extracted from the transmittance (T) spectrum of ML‐AgBiP_2_Se_6_ at 300 K (see inset), which exhibits a strong linear region starting from ≈1.4 eV to 1.5 eV, consistent with an indirect allowed transition as the other indirect semiconductors.^[^ ^37^, ^38^, ^39^, ^40^ ^]^ The inset in Figure 2c shows the transmittance curves of ML‐AgBiP_2_Se_6_ at 300 K and 20 K near band edge. The hollow circles in Figure 2c represent the experimental (𝛼hν)^1/2^ data and the black solid lines are the linearly fitted results including phonon‐absorption and phonon‐emission processes appropriate for the phonon‐assisted indirect allowed transitions. For a single phonon process, the fitted formula of the absorption coefficient of a semiconductor can be expressed by:^[^ ^37^ ^]^

(2) $$\alpha h\nu =A_{\alpha_{\mathrm{photon},\mathrm{phonon}}}+B_{\alpha_{\mathrm{photon}}+e_{\mathrm{phonon}}}$$

and

(3) $$A_{\alpha_{\mathrm{photon},\mathrm{phonon}}}=\frac{C_{1}{\left(\mathrm{hv}-\left(E_{g}-E_{p}\right)\right)}^{2}}{e^{\frac{E_{p}}{\mathrm{kT}}}-1}$$

and

(4) $$B_{\alpha_{\mathrm{photon}}+e_{\mathrm{phonon}}=}\frac{C_{2}{\left(\mathrm{hv}-\left(E_{g}-E_{p}\right)\right)}^{2}}{1-e^{\frac{-E_{p}}{\mathrm{kT}}}}$$

where hν is the photon energy in eV, $A_{\alpha_{\mathrm{photon},\mathrm{phonon}}}$ corresponds to photon and phonon absorption and $B_{\alpha_{\mathrm{photon}}+e_{\mathrm{phonon}}}$ corresponds to photon absorption and phonon emission, with C_1_ and C_2_ are constants, and E_g_ is the indirect‐gap energy and E_p_ represents the averaged phonon energy. By eliminating the background absorption beforehand, E_g_ and E_p_ can be extracted from the spectral analysis using the above Equations (2)–(4). The indirect‐gap values (E_g_) of ML‐AgBiP_2_Se_6_ obtained from the optical‐absorption analysis are 1.46 and 1.601 eV at 300 and 20 K, respectively. The experimental indirect bandgap (E_g_) is close to the calculated value of single‐layer AgBiP_2_Se_6_ of 1.483 eV [i.e. $E_{1}^{\mathrm{ind}}$ at Γ‐K (VBM) to Γ point (CBM) transition] obtained from the band‐structure calculations as shown in Figure 2a. However, even the quasi‐direct band nature of multilayer AgBiP_2_Se_6_ remains unchanged, the calculated indirect and direct bandgaps done by DFT are still lower than those of the experimental values of optical measurements. From optical transmittance, the indirect bandgap ($E_{1}^{\mathrm{ind}}$) in ≈1.46 eV is slightly difficult to detect in standard absorption but emerges via derivative analysis using ΔT and (αhν)^1/2^ (as shown in Figure S7b,d, Supporting Information), including the detail temperature‐dependent transmittance and absorption spectra (Figure S7a,c, Supporting Information). The linear fitting parameters of (αhν)^1/2^ vs. hν for ML‐AgBiP_2_Se_6_ obtained from Figure S7d (Supporting Information) are listed in Table S4 (Supporting Information). The relatively lower indirect bandgap (≈1.46 eV) than that of the calculation value (1.483 eV) is due to a larger thickness of the measured multilayer (or bulk) sample. In 2D semiconductors, the bandgap generally decreases as the number of layers increases due to the enhancement of interlayer interactions. In a monolayer form, the electronic structure is dominated by strong quantum confinement and limited dielectric screening, resulting in a much wider bandgap. Additionally, from the fitting process in Equ. (2)‐(4), the value of average phonon energy E_p_ is 47 ± 12 meV, similar to the other indirect semiconductor with a layered structure.^[^ ^38^ ^]^

Figure 2d shows the µPL spectra of ML‐AgBiP_2_Se_6_ with various thicknesses from 55 nm (≈78 layers), 71 nm (≈111 layers) to 133 nm (≈190 layers), relates to the direct transition energy ($E_{2}^{d}$) of light emission as observed from the band‐structure calculations in Figure 2a. The PL peak positions of the three thickness portions in one ML‐AgBiP_2_Se_6_ (see Figure S8b–d, Supporting Information) are matched to locate at $E_{2}^{d}$ (FX)≈1.535 eV at 300 K. The sample image and thickness information for the three parts of ML‐AgBiP_2_Se_6_ are identified by AFM and shown in Figure S8a–d (Supporting Information) for comparison. The strong photoluminescence observed in multilayer AgBiP_2_Se_6_ can be attributed to its quasi‐direct band gap. In a quasi‐direct semiconductor, the energy difference between the indirect and direct band edges is very small, allowing electrons to recombine radiatively at the direct band edge with high probability. This leads to efficient light emission while still retaining some characteristics of an indirect gap, which reduces nonradiative losses. Therefore, even in multilayer samples, AgBiP_2_Se_6_ exhibits strong PL, highlighting its potential for optoelectronic applications. This situation can also be found in the other quasi‐direct layered chalcogenide of ZrS_3_.^[^ ^9^ ^]^ The occurrence of PL emission reveals a compelling indicator for the strong radiative recombination occurred inside a direct‐like semiconductor. This situation is interested for the direct evidence of a light‐emitting material. The observation of band‐edge PL is unusual in conventional indirect semiconductors (e.g., bulk Si), however it is typical for a quasi‐direct semiconductor, where the carriers could be thermally excited to the direct transition states and the related excitonic effect can assist in the radiative recombinations.^[^ ^3^, ^9^ ^]^ The absorption and thickness‐dependent PL results suggest a quasi‐direct band edge existed in the ML‐AgBiP_2_Se_6_ and the energy difference between the indirect and direct bandgaps is relatively small. The band‐structure variation related to the thickness change in the FE‐AgBiP_2_Se_6_ is minor and the thickness variation is insignificance to its band‐edge alteration.

### 2.3 Light Emission and Band‐Edge Transitions in Quasi‐Direct ML‐AgBiP_2_Se_6_ at Low Temperatures

**Figure 3:**
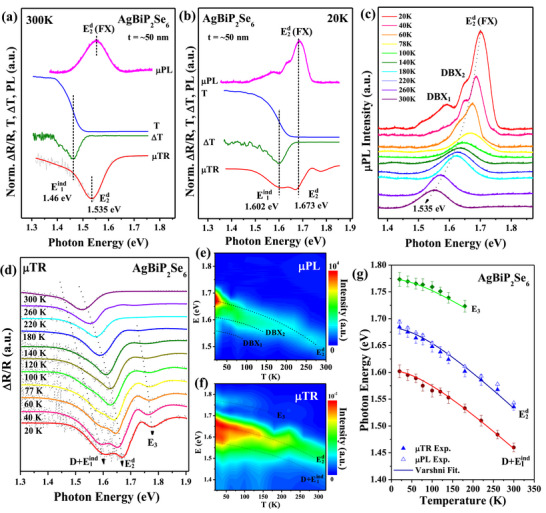
Optical properties of band‐edge transitions in FE‐AgBiP_2_Se_6_. The comparison of micro‐photoluminescence (µPL), transmittance (T), first derivative transmittane (ΔT), and micro‐thermoreflectance (εTR) spectra for the band‐edge transitions (including indirect and direct gap) of ML‐AgBiP_2_Se_6_ at a) 300 and b) 20 K. c) The temperature‐dependent µPL spectra at 20–300 K. d) The temperature‐dependent µTR spectra at 20–300 K. e) The 2D contour plot of µPL spectra of 20–300 K in ML‐AgBiP_2_Se_6_. f) The 2D contour plot of µTR spectra of 20–300 K in ML‐AgBiP_2_Se_6_. g) The analysis of temperature‐dependent transition energies of AgBiP_2_Se_6_ obtained by µPL and µTR. The solid lines are those of the Varshni fitting curves.

To further identify the indirect ($E_{1}^{\mathrm{ind}}$) and quasi‐direct $(E_{2}^{d}$) transitions in AgBiP_2_Se_6_, temperature‐dependent µPL, transmittance (T) and µTR measurements are carried out. **Figure** **3**a,b shows the comparison of optical measurements in ML‐AgBiP_2_Se_6_ at 300 and 20 K. The µPL and µTR are implemented to characterize the direct band‐edge transition^[^ ^41^ ^]^ while the transmittance is used for identification of the indirect bandgap.^[^ ^40^ ^]^ Figure 3a (Figure 3b) shows that at 300  and 20 K the indirect band edge in both transmittance and first‐derivative transmittance ΔT spectra that related to $E_{1}^{\mathrm{ind}}$ in Figure 2a lies below the direct band‐edge transition of $E_{2}^{d}$ measured by µPL and µTR experiments. From the energy difference of indirect ($E_{1}^{\mathrm{ind}}$) and direct $(E_{2}^{d}$) transitions (i.e. $E_{2}^{d}-{\;E}_{1}^{\mathrm{ind}}$ = 71 to 75 meV) observed in Figure 3a,b and in combination with the theoretical band‐structure calculations in Figure 2a, the indirect band nature and quasi‐direct band edge of the ferroelectric ML‐AgBiP_2_Se_6_ are confirmed. As shown in Figure 3a,b, the energy positions of $E_{1}^{\mathrm{ind}}$ and $E_{2}^{d}$ in a 50 nm thick AgBiP_2_Se_6_ measured by transmittance, µPL and µTR are all well matched at 300 and at 20 K. The transition energies are blue shifted as the temperature is lowered down from 300 down to 20 K to demonstrate the general semiconductor behavior.

Figure 3c shows the temperature‐dependent µPL spectra of ML‐AgBiP_2_Se_6_ from 20 to 300 K. At 300 K, the main emission originates from free exciton (denoted as FX) of FE‐AgBiP_2_Se_6_, which relates to the direct band gap ($E_{2}^{d}$) of AgBiP_2_Se_6_, appeared at 1.535 eV as a broadened spectral feature. Upon cooling to 20 K, the spectrum exhibits three distinct peaks, with the FX‐related emission becoming sharper, while the other two peaks are likely associated with donor (defect)‐bound excitonic recombination denoted as DBX, consisting of bound exciton complex (BEC) and donor‐acceptor pairs (DAP).^[^ ^42^, ^43^, ^44^ ^]^ The DBX_1_ located at ≈1.542 eV and DBX_2_ at ≈1.603 eV at 20 K may come from defect‐bound excitonic emissions in the ML‐AgBiP_2_Se_6_ layer crystal. The inference of origins of the DBX and FX emissions may be verified by the degradation speed of the PL intensity with the increase of temperatures. As shown in Figure 3c, the emission peaks of the DBX_1_ and DBX_2_ degraded very fast at 20–78 K due to their free‐to‐bound behavior from the donor (defect) sites and finally they will become free excitons to merge into the main FX emission observed from 78 to 300 K. This is why the degradation speed of the $E_{2}^{d}$ (FX) intensity is slower than those of the DBX_1_ and DBX_2_ as displayed in Figure 3c from 20 to 300 K.

To certify the quasi‐direct band‐edge transitions of ML‐AgBiP_2_Se_6_ observed in transmittance and µPL, µTR experiments are implemented. The temperature‐dependent µTR spectra from 300 down to 20 K are presented in Figure 3d in the photon energy range between 1.3 eV and 1.9 eV. The direct band edge ($E_{2}^{d}$) appeared in ≈1.53 eV at 300 K and shifts to ≈1.673 eV at 20K. There are three transition features detected at 20 K. The broadened feature below $E_{2}^{d}$ indicates the indirect combined with defect‐related transition and become sharper at 20 K in ≈1.6 eV, denoted as D+$E_{1}^{\mathrm{ind}}$, such as previously mentioned by a quasi‐direct multilayered ZrS_3_.^[^ ^9^ ^]^ The other exciton (E_3_) appears from ≈180 K and become enhanced at 20 K, correlating with the higher‐level excitonic transition related to $E_{2}^{d}$ near band edge at lower temperatures. Figure 3e,f shows the 2D contour plots of the emission and absorption spectra of the band edge transitions in µPL and µTR, respectively, to see the peak‐intensity profile mapping. Essentially, the maximum intensified exciton is $E_{2}^{d}$ (FX), which positions at 1.673 eV at 20 K (also see Figure 3b) to dominate the main band‐edge emission of ML‐AgBiP_2_Se_6_ in the whole temperature range. The temperature dependence of transition energy in Figure 3e,f, fitted using Varshni fitting (parameters listed in Table S5, Supporting Information), is shown in Figure 3g. The solid symbols with error bars represent the experimental data of D+$E_{1}^{\mathrm{ind}}$ (red), $E_{2}^{d}$ (blue) and E_3_ (green) from µTR while the hollow blue rectangles represent the experimental data of $E_{2}^{d}\;$ in µPL derived from Figure 3c,d, and the solid lines correspond to the least‐square fits of Varshni empirical formula: E_i_(T) = E_i_(0)‐(α_i_⋅T^2^)/(β_i_+T), where i denotes the respective transition, E_i_(0) represents the energy at absolute zero (0 K), α_i_ indicates the strength of the electron (or exciton)–phonon interaction, and β_i_ is a parameter closely associated with Debye temperature. From the Varshni fits, the obtained Debye temperature is determined to be 250 ± 20 K in this material. The values of exciton‐phonon coupling strength in the indirect and direct related transitions D+$E_{1}^{\mathrm{ind}}$, $E_{2}^{d}$ and E_3_ are shown to be similar and within α_i_ = (0.8 ± 0.1) × 10^−3^ eV K^−1^. This result indicates the temperature coefficients of lattice shrinkage and dilation along Γ‐K (indirect) direction and at the Γ point (direct) are resembled (see the band structure in Figure 2a) to support the direct‐like optical behavior in the quasi‐direct ML‐AgBiP_2_Se_6_ which possessing an indirect‐band nature.

### 2.4 Fabrication of a Vertically‐Stacked Heterojunction Solar Cell by p‐Ga_0.5_In_0.5_Se/n‐AgBiP_2_Se_6_ Van Der Waals Heterostructure

**Figure 4:**
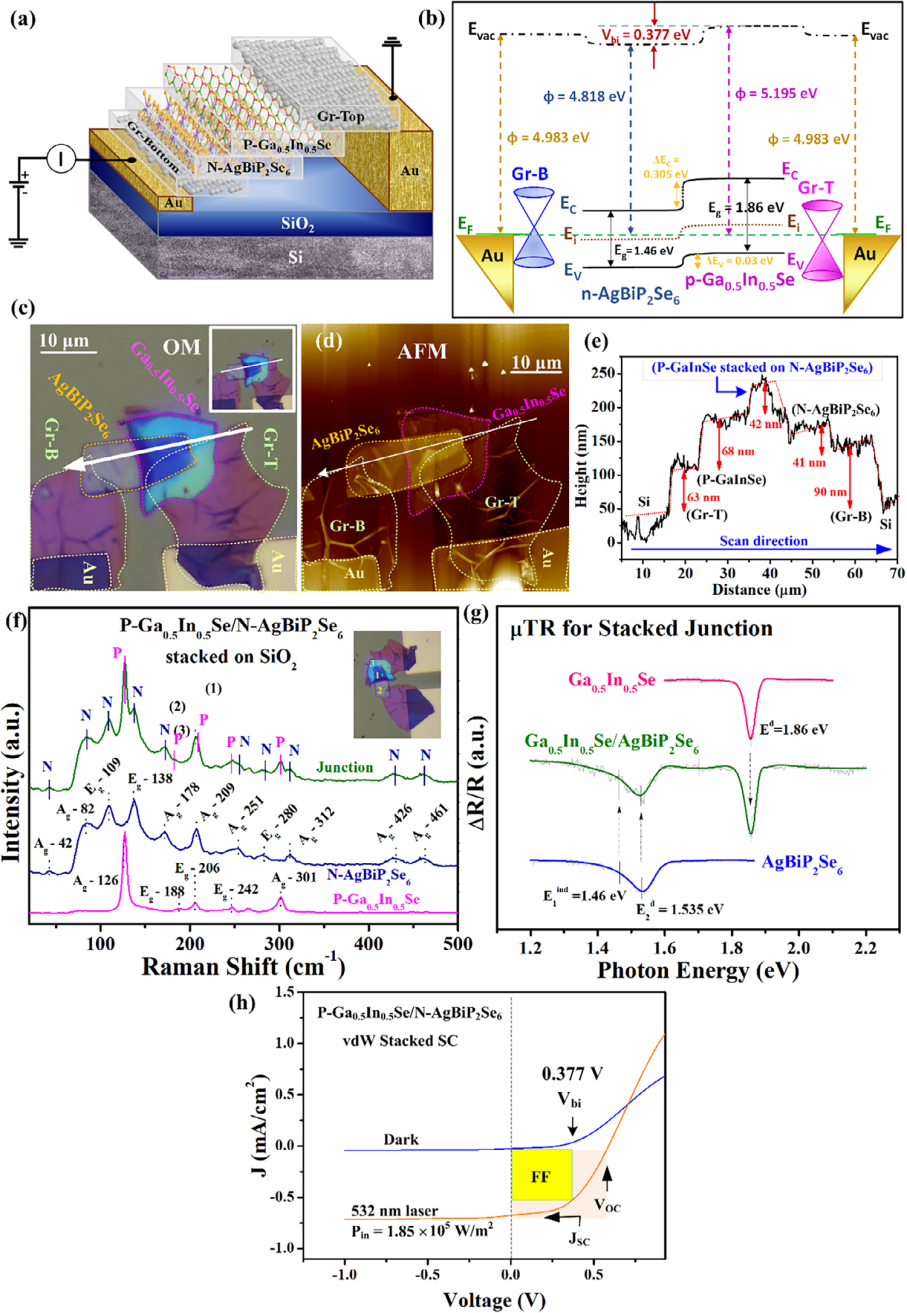
Fabrication, electrical and optical properties of the *p*‐Ga_0.5_In_0.5_Se/*n*‐AgBiP_2_Se_6_ heterojunction solar cell (SC). a) Schematic diagram and operation of the stacking SC. b) Energy band diagram of the stacked heterojunction SC at thermal equilibrium. The images of c) optical microscope (OM) and d) AFM of the stacking SC. e) The thickness distribution of the stacked heterojunction SC obtained from AFM. f) The µRaman spectra and g) µTR spectra of the junction area, Ga_0.5_In_0.5_Se, and AgBiP_2_Se_6_ in the p‐Ga_0.5_In_0.5_Se/n‐AgBiP_2_Se_6_ heterojunction to identify the materials’ profile. h) The *J*–*V* characteristic of the rectified *p*‐Ga_0.5_In_0.5_Se/*n*‐AgBiP_2_Se_6_ heterojunction SC under dark and under illumination condition of green laser. The curve under dark condition shows a built‐in potential (*V*_bi_) of ≈0.377 V in the SC.

To further explore the applications of ferroelectric multilayer AgBiP_2_Se_6_ with its quasi‐direct bandgap nature, a vertically stacked heterojunction was fabricated by stacking p‐type Ga_0.5_In_0.5_Se on n‐type AgBiP_2_Se_6_. **Figure** **4**a illustrates a schematic of the prototype device, which is constructed by vertically stacking p‐type Ga_0.5_In_0.5_Se and n‐type AgBiP_2_Se_6_ nanoflakes on a Si/SiO_2_ substrate. Graphene and patterned Au contacts serve as ohmic‐contact electrodes to form a p–n junction solar cell (SC). Such kind of van der Waals (vdW) heterostructure benefits from the absence of dangling bonds at the interface, enabling smooth junction and atomically sharp interfaces by using graphite (graphene) as the ohmic‐contact layers.^[^ ^45^ ^]^ The use of Ga_0.5_In_0.5_Se layer is owing to its direct bandgap (shown in Figure S9b, Supporting Information), high photoresponse (shown in Figure S13d, Supporting Information), p‐type behavior,^[^ ^42^ ^]^ and exhibit high carrier concentration (hole) similar to AgBiP_2_Se_6_ (electron) that will be observed more in Table S6 (Supporting Information). The Ga_0.5_In_0.5_Se layered crystal was also grown by the CVT method similar to the other transition‐metal dichalcogenides.^[^ ^46^ ^]^ Figure S9a,b (Supporting Information) shows the direct bandgap of a ML‐Ga_0.5_In_0.5_Se (50‐nm thick) is around 1.86 eV obtained from transmittance, µPL and µTR measurements at RT. For the stacking device, Figure 4b depicts the equilibrium band diagram of the p‐Ga_0.5_In_0.5_Se/n‐AgBiP_2_Se_6_ stacked heterojunction SC (i.e. graphene and Au as the ohmic‐contact electrodes due to the flexibility, transparent and closely‐contact characteristics of graphene material) at zero‐bias condition. It reveals a typically type‐II heterostructure band alignment with both CBM and VBM of the ML p‐Ga_0.5_In_0.5_Se lying above the corresponding CBM and VBM of the ML n‐AgBiP_2_Se_6_ for resulting in a staggered heterojunction with valence‐band discontinuity (ΔE_V_) and conduction‐band discontinuity (ΔE_C_) existed in the junction area. In the p‐Ga_0.5_In_0.5_Se/n‐AgBiP_2_Se_6_ type‐II heterojunction, the built‐in electric field is directed from the n‐type AgBiP_2_Se_6_ toward the p‐type Ga_0.5_In_0.5_Se across the depletion region. This internal field drives photoexcited electrons toward the n‐side and holes toward the p‐side, thereby spatially separating the electron–hole pairs. Such separation reduces recombination at the interface and facilitates efficient charge transport, which is essential for the photovoltaic performance of the stacking device.

To characterize the band diagram of the p‐Ga_0.5_In_0.5_Se/n‐AgBiP_2_Se_6_ heterojunction in detail, Kelvin probe work‐function measurement and X‐ray photoelectron spectroscopy (XPS) of the two layered materials are performed. Figure S10a–d, Supporting Information) respectively reveal the work‐function distribution histogram and mapping profile on a 750×750 µm^2^ area of the AgBiP_2_Se_6_ (Ga_0.5_In_0.5_Se) layered crystal. It is clearly that the work functions (ϕ) of the as‐grown ML n‐AgBiP_2_Se_6_ and ML p‐Ga_0.5_In_0.5_Se are uniformly distributed and are centered at 4818 and 5195 meV, respectively. Figure S10e (Supporting Information) shows the measured work functions in the consisted materials of metal (Au/Ti, ϕ = 4983 meV), graphene (Gr, ϕ = 5078 meV), n‐AgBiP_2_Se_6_ and p‐Ga_0.5_In_0.5_Se for verification of the ohmic‐contact behavior of electrodes in the p‐Ga_0.5_In_0.5_Se/n‐AgBiP_2_Se_6_ stacked heterojunction SC. Figure S10f (Supporting Information) displays the separated band diagram of the individual materials by n‐AgBiP_2_Se_6_ and p‐Ga_0.5_In_0.5_Se with the energy values obtained from the optical bandgap measurement, Kelvin‐probe work function, and XPS result. The energy difference between the VBM and Fermi level (E_F_) in the layered n‐AgBiP_2_Se_6_ and p‐Ga_0.5_In_0.5_Se can be determined by the linear extrapolation of the VB XPS spectra as displayed in Figures S11b and S12b,c (Supporting Information), and the obtained values of E_F_‐VBM are 0.82 eV for n‐AgBiP_2_Se_6_ and 0.413 eV for p‐Ga_0.5_In_0.5_Se, respectively. The bandgap of AgBiP_2_Se_6_ is E_1_^ind^ = 1.46 eV and direct gap of Ga_0.5_In_0.5_Se is E^d^ = 1.86 eV, which means the values of ΔE_C_ and ΔE_V_ in the heterojunction are 0.305 and 0.03 eV as displayed in Figure S10f (Supporting Information). The values of electron affinity (qχ) of AgBiP_2_Se_6_ and Ga_0.5_In_0.5_Se are thus determined to be 4.178 and 3.748 eV. For XPS study, the full spectrum of AgBiP_2_Se_6_ is shown in Figure S11a (Supporting Information) and the XPS results of Ag 3*d*, Bi 4*f*‐Se 3*p*, P 2*p*‐Se, and Se 3*d* peaks are exactly shown in S11c‐S11f for identification of high crystallinity of the ML‐AgBiP_2_Se_6_. Moreover, the full XPS spectrum, Ga 2*p*, In 3*d*, and Se 3*d* orbitals of the ML‐Ga_0.5_In_0.5_Se are also shown in S12a and S12d‐S12f for demonstration of high crystal quality of the stacked Ga_0.5_In_0.5_Se consisted in the heterojunction SC.

Though all experiments of work function, optical bandgap and EF‐VBM of XPS have been implemented, the real equilibrium band diagram of p‐Ga_0.5_In_0.5_Se/n‐AgBiP_2_Se_6_ stacked heterojunction SC is depicted in Figure 4b. The built‐in potential can be determined to be V_bi_ ≈ 0.377 V from the work function difference of obtained in Figure S10e (Supporting Information) across a space‐charge region at the p‐n junction. The related electrical properties of layered AgBiP_2_Se_6_ and Ga_0.5_In_0.5_Se are listed in Table S6 (Supporting Information), which indicates the Hall mobility are 131 and 244 cm^2^/V‐s for n‐AgBiP_2_Se_6_ and p‐Ga_0.5_In_0.5_Se, and their electron and hole concentrations are respectively 4.16 × 10^13^ and 3.36 × 10^13^ cm^−3^. In this case, maintaining a comparable carrier concentration on both sides facilitates the formation of a well‐balanced depletion region for achieving a larger photon‐absorption area for generation of electron‐hole pairs. This design helps carriers cross the heterojunction interface more easily, improves charge separation, and can enhance the short‐circuit current density (J_SC_), filling factor (FF) and photoelectric conversion efficiency (PCE). Figure 4c shows the microscope (OM) image of the real p‐Ga_0.5_In_0.5_Se/n‐AgBiP_2_Se_6_ stacked heterojunction diode with Au/Ti and graphene layers (Gr‐T: top layers and Gr‐B: bottom layers) as the ohmic‐contact electrodes. The corresponding AFM image of the heterojunction device is shown in Figure 4d. The white arrow lines in Figure 4c,d indicates the scan direction for the thickness‐profile measurement of the stacked heterojunction device and the thickness distribution profile is depicted in Figure 4e for illustration. It is clearly that the values of thickness are ≈68 nm for p‐Ga_0.5_In_0.5_Se and ≈42 nm for n‐AgBiP_2_Se_6_ in the stacked heterojunction. To identify the materials properties of the vertically stacked heterojunction device, µRaman and µTR measurements of each material region of p‐Ga_0.5_In_0.5_Se (red‐pink color spectra in area 3, see the inset of Figure 4f), n‐AgBiP_2_Se_6_ (dark‐blue color spectra, area 2) and the stacked junction (green color spectra, area 1) in the stacked device were implemented and the results are respectively shown in Figure 4f,g. For the Raman results, the Raman peaks denoted as A_g_‐126, E_g_‐188, E_g_‐206, E_g_‐242, and A_g_‐301 cm^−1^ of P‐Ga_0.5_In_0.5_Se (red‐pink color) in Figure 4f are corresponding to the in‐plane and out‐of‐plane vibration modes similar to those observed in the hexagonal GaSe and GaSe_1‐x_S_x_ III‐VI layers.^[^ ^42^ ^]^ The Raman spectrum and peak modes of n‐AgBiP_2_Se_6_ (dark‐blue color) are matched well with those observed in Figure 1d. For the Raman peaks of the junction part (green‐color spectrum), a combination of the peak modes containing P‐Ga_0.5_In_0.5_Se (denoted as “P”) and N‐AgBiP_2_Se_6_ (denoted as “N”) is clearly demonstrated for proving a well‐behaved stacking structure for the heterojunction SC. Measurement of optical transitions can be served as the best performance evaluation for assessment of the vertically stacked heterojunction device. Figure 4g shows the µTR spectra of P‐Ga_0.5_In_0.5_Se (red‐pink color), N‐AgBiP_2_Se_6_ (dark‐blue color) and the junction part (green color). The direct gap of Ga_0.5_In_0.5_Se is positioned at 1.86 eV, similar to that in Figure S9b (Supporting Information). The indirect and direct bandgaps of the quasi‐direct ML‐AgBiP_2_Se_6_ is E_1_^ind^ = 1.46 eV and E_2_^d^ = 1.535 eV, matching well with those observed in Figure 3a. The stacked junction part of P‐Ga_0.5_In_0.5_Se on N‐AgBiP_2_Se_6_ reveals a perfect combination of the optical transitions (green‐color spectrum) as detected in both layered materials shown in Figure 4g.

Prior to study the opto‐electrical behavior of the heterojunction SC, the photoconductive responses of the n‐AgBiP_2_Se_6_ and p‐Ga_0.5_In_0.5_Se have also been evaluated. The representative schemes of photo current‐voltage (Photo I‐V) measurements for the layered AgBiP_2_Se_6_ and Ga_0.5_In_0.5_Se are depicted in Figure S13a,b (Supporting Information) for illustration. Where the silver paste was used as the contact medium of electrodes and the measurement results of Photo I‐V responses are demonstrated in Figure S13c (Supporting Information) for AgBiP_2_Se_6_ and presented in Figure S13d (Supporting Information) for Ga_0.5_In_0.5_Se, respectively. The Photo I‐V measurements are implemented under three different illumination conditions of dark, LED (white‐light light‐emitting diode), and tungsten halogen lamp (THL). The emission spectra of LED (blue) and THL (red) together with the bandgap positions of AgBiP_2_Se_6_ and Ga_0.5_In_0.5_Se are shown in the inset in Figure S13c,d (Supporting Information) for contrast. It is clearly that the bandgap of AgBiP_2_Se_6_ matches with the maximum response of THL spectrum (≈1.5 eV) while the direct bandgap of Ga_0.5_In_0.5_Se agrees well with the LED peak intensity in the red‐light portion (≈2 eV). Thus layered AgBiP_2_Se_6_ got the highest photocurrent with the THL illumination (see Figure S13c, Supporting Information) while bulk Ga_0.5_In_0.5_Se obtained the highest photocurrent response under LED's illumination (see Figure S13d, Supporting Information). All the measurement results of photoconductivity obtained from Photo I‐V in Figure S13c,d (Supporting Information) are listed in Table S7 (Supporting Information) for comparison. Essentially AgBiP_2_Se_6_ dominates the optical absorption in the near‐infrared (NIR) region while Ga_0.5_In_0.5_Se handles the generation of photo carriers in the visible range. The optimum design of a stacked p‐Ga_0.5_In_0.5_Se/n‐AgBiP_2_Se_6_ heterojunction SC becomes more pronounced when considering the operation mechanism of a p‐n heterojunction SC with the absorption range from NIR to visible light.

Figure 4h shows the performance of current density versus voltage (*J–V*) curves of the p‐Ga_0.5_In_0.5_Se/n‐AgBiP_2_Se_6_ vdW stacked heterojunction SC measured under dark and illumination conditions. The light source is a 532‐nm solid state laser with incident power density adjusted to P_in_ = 1.85 × 10^5^ W⋅m^−2^ (1.85 × 10^1^ W⋅cm^−2^) onto the junction part of the stacked heterostructure. The *J–V* characteristics under dark condition reveal rectified behavior with a built‐in potential of V_bi_≈0.377 V in the p‐Ga_0.5_In_0.5_Se/n‐AgBiP_2_Se_6_ stacked p‐n junction diode. With consideration of the valence‐band discontinuity of ΔE_V_ = 0.03 eV (see Figure 4b), the start‐on voltage of the diode under forward bias is approximately at 0.407 V for rapid increasing the current as shown in Figure 4h under dark condition. We also identify the diode characteristic under dark condition to present an ideality factor (η) of ≈1.34 as shown in Figure S14 (Supporting Information). As shown in Figure 4h, under the illumination of a 532‐nm laser, the vdW stacked heterojunction SC reveals a clear and significant photovoltaic response caused by photo generated carriers and also efficient separation of the electron‐hole pairs through built‐in electric field of the depletion region of hetrojunction. The J‐V characteristic of SC is similar to previous vdW stacked photovoltaic device made by a GaSe/InSe heterojunction.^[^ ^47^ ^]^ From the photovoltaic response depicted in Figure 4h, an open‐circuit voltage (V_OC_) of ≈0.58 V, short‐circuit current density (J_SC_) of ≈0.73 mA cm^−2^, a fill factor (FF) of 63% and a photoelectric conversion efficiency (PCE) of ≈0.583% are obtained from the p‐Ga_0.5_In_0.5_Se/n‐AgBiP_2_Se_6_ vdW stacked heterojunction SC. The filling factor can be evaluated as FF = P_M(ele)_/(J_SC_×V_OC_) and the photoelectric conversion efficiency is estimated by PCE = P_M(ele)_/P_in(opt)_ (×100) %. Where P_M(ele)_ is the maximum electric power (J × V) defined as the maximum rectangular area limited by the fourth quadrant of the J‐V curve under the illuminated condition and P_in(opt)_ represents the incident optical power density. The obtained parameters of photoelectric conversion of the p‐Ga_0.5_In_0.5_Se/n‐AgBiP_2_Se_6_ stacked heterojunction SC of J_SC_, V_OC_, FF, and PCE etc. are listed in **Table** **1** together with the other heterojunction SCs as p‐MoTe_2_/n‐MoS_2_,^[^ ^48^ ^]^ p‐GaSe/n‐MoSe_2_,^[^ ^49^ ^]^ and p‐GaSe/n‐ZrS_3_^[^ ^9^ ^]^ (i.e. using a 532‐nm laser as the excitation source) are also included for comparison. Among them, the present p‐Ga_0.5_In_0.5_Se/n‐AgBiP_2_Se_6_ vdW stacked heterojunction shows the best PCE ≈ 0.583%, maybe owing to the highest FF value of ≈63% and the unique quasi‐direct band nature of ML‐AgBiP_2_Se_6_ for simultaneously absorbing photons by indirect and direct bandgaps (see Figure 4g). Moreover, the formation of type II heterostructure in the p‐Ga_0.5_In_0.5_Se/n‐AgBiP_2_Se_6_ junction (see Figure 4b) can also promote additional optical transitions from the VBM of p‐Ga_0.5_In_0.5_Se across to transit to the CBM of the n‐AgBiP_2_Se_6_ for improving PCE in the stacked solar cell. The concept of type II and type I heterostructures and quantum wells of layered materials can be further implemented by using epitaxial‐growth method like the fabrication of traditional III‐V GaAs and InGaAs devices^[^ ^50^ ^]^ for future nanoelectronics and optoelectronics applications.

**Table 1:** The comparison of short‐circuit current density (J_SC_) generated under illumination condition, open‐circuit voltage (V_OC_), fill factor (FF), and the photoelectric conversion efficiency (PCE) in some stacked p‐n junction SCs made by layered materials.

Junction	t [nm]	Light Source, λ [nm]	J_SC_ [mA/cm^2^]	V_OC_ [V]	P_in(opt)_ [W⋅cm^−2^]	E_g_ [eV]	FF [%]	PCE, *η* [%]	Refs.
**p‐α‐MoTe_2_/n‐MoS_2_**	2.2 (3L)	Laser 532 nm	0.22	0.3	N/A	1/1.8	N/A	0.45	[48]
**p‐GaSe/n‐MoSe_2_**	≈79–118	Laser 532 nm	3.11	≈0.4	0.1	2/1.6	44	0.54	[49]
**p‐GaSe/n‐ZrS_3_** **(E//b)**	70 / 25	Laser 532 nm	1.454	1	1.88 × 10^1^	1.94/1.992	53	0.412	[9]
**BPV AgBiP_2_Se_6_**	4.7	Laser 532 nm	5.7	0.09	0.1	1.5	25	0.13	[21]
**p‐In_0.5_Ga_0.5_Se/n‐AgBiP_2_Se_6_**	68 / 42	Laser 532 nm	0.73	0.581	1.85 × 10^1^	1.86/1.46	63	0.583	This work

### 2.5 Quasi‐Direct ML‐AgBiP_2_Se_6_ Acted as an Excellent Photocatalyst for Environmental Protection

**Figure 5:**
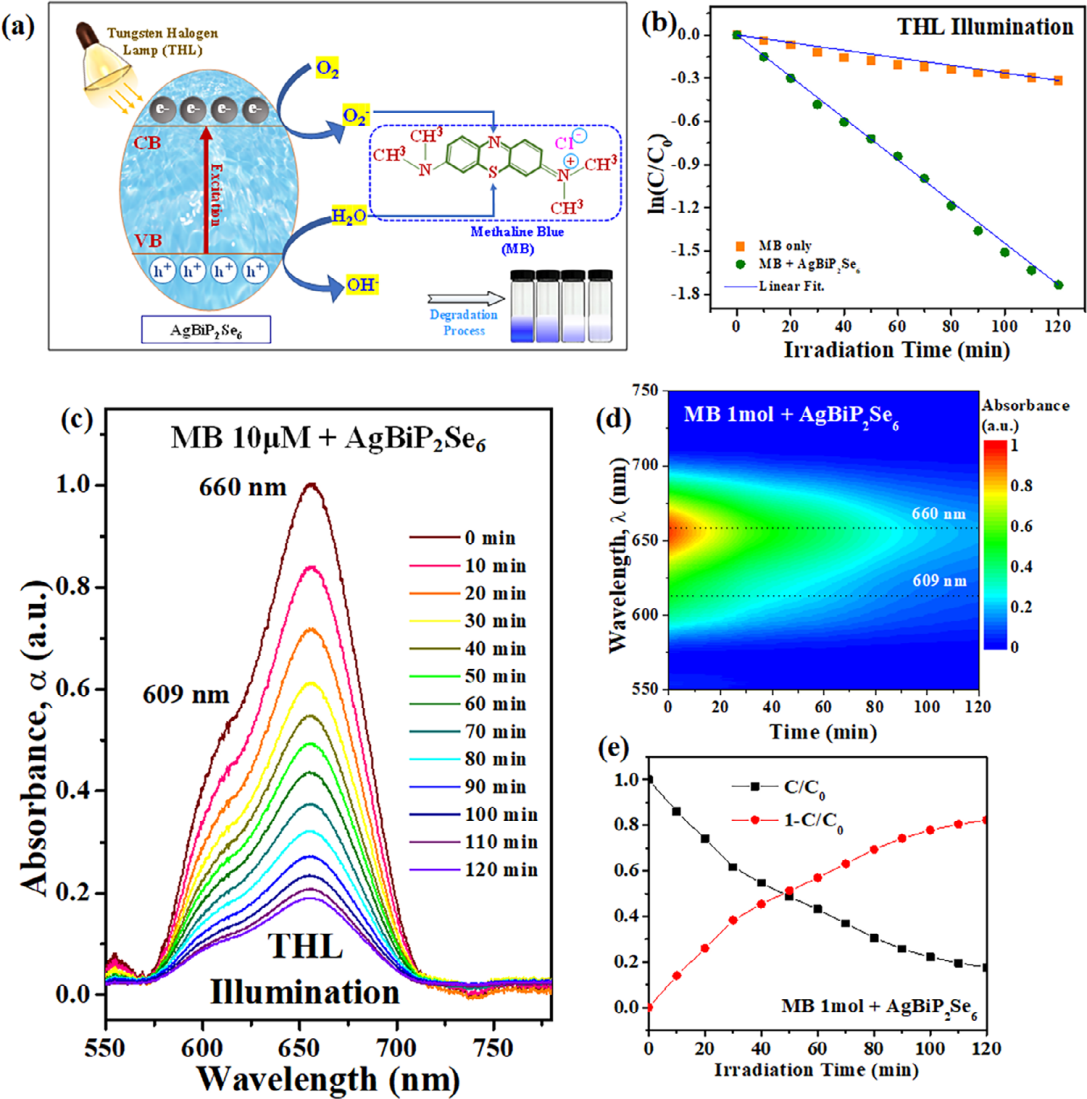
Photodegradation characteristic of ML‐AgBiP_2_Se_6_. a) Schematic diagram of photocatalytic process using methylene blue (MB) as the pollutant with tungsten halogen lamp (THL) as the light source. b) The degradation rate of AgBiP_2_Se_6_ in MB compared to MB without AgBiP_2_Se_6_. c) Photodegradation spectra of MB solution by AgBiP_2_Se_6_ photocatalyst under THL illumination. d) The 2D contour‐plot spectra in each wavelength peak of MB with AgBiP_2_Se_6_ nanoflakes degradation. e) The plot of degraded efficiency (C/C_0_) and undegraded efficiency (1‐C/C_0_) versus time for the MB solution with AgBiP_2_Se_6_ as the photocatalyst.

Owing to quasi‐direct AgBiP_2_Se_6_ may efficiently absorb photons by the indirect and direct gaps to generate photo carriers, the photocatalytic activity of AgBiP_2_Se_6_ flakes was systematically evaluated through the degradation of methylene blue (MB) solution under visible‐light irradiation by using a tungsten halogen lamp (THL) as the illumination light source. As illustrated in the schematic diagram in **Figure** **5**a, the layered structure of AgBiP_2_Se_6_ facilitates efficient generation and separation of photogenerated charge carriers under THL illumination. The absorbed photons promote electrons (e^−^) from the valence band (VB) to the conduction band (CB), producing electron–hole pairs that migrate to the surface of the flakes. The surface defects, intrinsic electric fields due to ferroelectric polarization, and edge states in multilayer AgBiP_2_Se_6_ can facilitate photocatalytic function to the MB degradation. Moreover, the liquid and semiconductor junction formed at the interface of MB solution and AgBiP_2_Se_6_ nanosheets can naturally construct a band‐bending space‐charge region with built‐in electric field, and which will efficiently separate the photo‐generated electron‐hole pairs for contribution to the photocatalytic behavior. These photogenerated charge carriers may migrate to the catalyst surface where they participate in redox reactions: (e^−^ + O_2_ → •O_2_^−^) and (h^+^ + H_2_O → •OH). These reactive oxygen species such as superoxide (•O_2_^−^) and hydroxyl radicals (•OH) play a crucial role in degrading organic pollutants (such as methylene blue MB dyes) into non‐toxic by products. The photocatalytic degradation kinetics of MB, monitored by absorption spectroscopy, reveal a pronounced enhancement in degradation rate in the presence of ML‐AgBiP_2_Se_6_ compared to that of without AgBiP_2_Se_6_ in the MB solution (i.e. the degradation spectra are shown in Figure 5c,d and rate‐constant analysis is displayed in Figure 5b). The photodegradation rate constant is calculated using the pseudo‐first‐order reaction, defined as ln ($\frac{C}{C_{0}})=-k\cdot t$,^[^ ^51^ ^]^ where C_0_ denotes the initial concentration, C represents the concentration degraded over time t, and k corresponds to the rate constant in photocatalytic reaction. The time‐dependent plots of ln(C/C_0_) versus irradiation time present ML‐AgBiP_2_Se_6_ exhibits significantly faster degradation speed (k = 1.5 × 10^−2^ min^−1^) than MB (k = 5 × 10^−4^ min^−1^), demonstrated by a steeper linear slope in Figure 5b. This behavior attributed to higher surface area of multilayer compared to bulk, improved charge separation and carrier mobility in 2D systems, also in stronger interaction with visible light due to reduced dimensionality. As shown in Figure 5e, the cross point of the (C/C_0_) and (1‐C/C_0_) versus time curves also indicates the half‐lifetime degradation cycle of the MB dyes by AgBiP_2_Se_6_ photocatalyst is about 48 min. Moreover, the degradation rate (k) of AgBiP_2_Se_6_ is shown faster than that of the other layered chalcogenides of SnS_2_ (k = 7.4 × 10^−4^ min^−1^) and WS_2_ (k = 8.3 × 10^−4^ min^−1^)^[^ ^52^ ^]^ as well as much quicker than the cubic sulfides of ZnS (k = 1.09 × 10^−3^ min^−1^) and CdS (k = 2.98 × 10^−3^ min^−1^)^[^ ^53^ ^]^ for photodegradation of the MB pollutant dyes under the white‐light illuminations. The related results are listed in Table S8 (Supporting Information) for contrast. In comparison, other chalcogenides such as WS_2_ (1.35–2.05 eV), SnS_2_ (2.18–2.41 eV), and ZnS (≈3.6 eV) either absorb less visible light or have faster recombination, explaining the higher degradation efficiency of AgBiP_2_Se_6_. Compared with these layered materials, AgBiP_2_Se_6_ combines a small bandgap for visible light absorption with a quasi‐direct nature that slows recombination and contributes to its high degradation efficiency (82.5%). These findings also indicate that ML‐AgBiP_2_Se_6_ is a promising candidate for solar‐driven photocatalysis in environmental remediation applications, especially for the removal of persistent organic dye pollutants in the wastewater systems.

## 3 Conclusion

High‐quality layered AgBiP_2_Se_6_ has been synthesized and grown by CVT. ML‐AgBiP_2_Se_6_ has been identified as a quasi‐direct bandgap nature, combining the optical advantages of direct semiconductors with the electronic structure of an indirect bandgap. This is evidenced by the small energy difference (≈0.075 eV) between indirect of ≈1.46 eV and direct (≈1.535 eV) transitions from the band structure calculation and optical measurements. This near‐degeneracy allows AgBiP_2_Se_6_ to retain the strong optical response typically associated with direct bandgap semiconductors, as confirmed by pronounced micro‐photoluminescence (µPL), micro‐thermoreflectance (µTR), and efficient light absorption in the NIR to visible range. Temperature‐dependent µPL and µTR measurements further reveal a blue‐shifting band edge and enhanced excitonic stability at cryogenic temperatures, reinforcing the presence of strong excitonic interactions in the 2D layered system.

The strong absorption in the visible region, combined with a high exciton binding energy, suggests AgBiP_2_Se_6_ is well‐suited for light‐harvesting applications. To explore this potential, we fabricated a vertically‐stacked heterojunction between n‐type AgBiP_2_Se_6_ and p‐type Ga_0.5_In_0.5_Se. The resulting device exhibits a clear photovoltaic response under illumination condition, with open‐circuit voltage and short‐circuit current characteristics indicative of an effective type‐II band alignment. Notably, the clean interface achieved through dry transfer methods facilitates efficient charge separation and transport across the junction, which is critical for practical device integration.

Beyond the photovoltaic usage, we also demonstrate that ML‐AgBiP_2_Se_6_ exhibits notable photocatalytic activity. Under visible‐light irradiation, the material efficiently degrades methylene blue (MB) dye in aqueous solution, outperforming several other 2D materials reported for similar applications. This performance is attributed to the favorable band edge positions straddling the redox potentials of water, in conjunction with the narrow bandgap and excellent carrier dynamics of this quasi‐direct system.

In summary, ML‐AgBiP_2_Se_6_ is a multifunctional semiconductor that merges the electronic benefits of an indirect gap with the optical and excitonic strengths of a direct‐gap material. Its unique combination of properties (room‐temperature ferroelectric, quasi‐direct band structure, strong light‐matter interaction, and environmental photocatalytic functionality) makes AgBiP_2_Se_6_ a promising candidate for integration into digital electronics, flexible photodetectors, ultrathin solar cells, and light‐driven environmental remediation platforms.

## 4 Experimental Section

### Growth of AgBiP_2_Se_6_ Layered Crystals

AgBiP_2_Se_6_ single crystals were synthesized and grown via chemical vapor transport (CVT) using ICl_3_ as the transport agent. High‐purity elements (Ag, Bi, P, Se; 99.99%) were mixed in a 1:1:2:6 molar ratio (10 g in total) with ICl_3_ (10 mg.cm^−3^), and then sealed in an evacuated quartz ampoule. The ampoule was cooled with liquid nitrogen and sealed at ≈10^−6^ Torr. Each material was synthesized at 760 °C for 48 h, followed by crystal growth in a 760 °C → 680 °C gradient over 288 h. Resulting crystals exhibited layered structures with area sizes up to ≈2 cm^2^ and thicknesses up to 300 µm. Weak interlayer van der Waals forces enabled mechanical exfoliation of the layer crystal onto SiO_2_/Si substrates using scotch tapes.

### Structural and Optical Characterization

X‐ray diffraction (XRD) measurements (Bruker D2 PHASER, Cu Kα, 10°–60°) confirmed the crystallinity of the samples. Lattice parameters were determined by high‐resolution transmission electron microscopy (HRTEM, Tecnai F20 G2), with selected‐area electron diffraction (SAED) performed on exfoliated flakes. X‐ray photoelectron spectroscopy (XPS, ULVAC‐PHI) was used to quantify the elemental composition. Micro‐Raman (µRaman) and micro‐photoluminescence (µPL) spectra were acquired using a dual‐laser (532/632.8 nm) RAMaker system equipped with polarizers and neutral density filters. Micro‐thermoreflectance (µTR) measurements^[^ ^6^ ^]^ were conducted using a 150 W halogen lamp dispersed by a monochromator (1200 groves per mm grating), and the signals were detected with an HUV2000B Si photodetector.

### DFT Calculations

First‐principles DFT calculations were performed using Quantum ESPRESSO. Electronic structure was computed with SSSP pseudopotentials and PBE‐GGA. A 33 Ry cutoff and Monkhorst‐Pack grids (9×9×1 for SCF, 30×30×1 for DOS) ensured convergence. Atomic relaxation used BFGS until forces <0.01 eV Å^−1^. A vacuum of 18 Å prevented periodic interactions. Ferroelectricity was modeled with atomic displacements (Z_Ag_  =  0.34 Å, Z_Bi_  =  −0.17 Å) and paraelectric phases retained symmetric positions.

### Electrical Characterization

Electrical measurements were performed using hot‐probe and photo current‐voltage (Photo *I–V*) techniques. Hall effect and resistivity were evaluated using the van der Pauw method (Keithley 6220/2182) under a magnetic field of 0.7 T. Temperature‐dependent resistivity (20–300 K) was measured with a Lakeshore 335 system. The work function of semiconductor (ϕ_s_) was determined using a Kelvin probe (SKP5050), calibrated against gold references (ϕ ≈ 5.1 eV).

### Device Fabrication of a p‐Ga_0.5_In_0.5_Se/n‐AgBiP_2_Se_6_ Stacked Heterojunction Solar Cell

Few‐layer and multilayer (ML) Ga_0.5_In_0.5_Se, AgBiP_2_Se_6_, and graphene were exfoliated and sequentially stacked onto Au‐patterned SiO_2_/Si substrates using a polydimethylsiloxane (PDMS)‐assisted transfer method under a microscope equipped with three‐axis micromanipulators. Atomic force microscopy (AFM) was used to confirm the layer thicknesses. Graphene served as a contact layer to facilitate ohmic behavior. Current – voltage (*J–V*) characteristics were measured using a Keithley 230/6485 instrumentation setup. Laser excitation at 532 nm and polarization‐dependent optical measurements were carried out using a RAMaker µRaman system with rotatable dichroic polarizers. The optical power of light source was monitored with an Ophir thermal‐sensor meter.

### Performance Evaluation of AgBiP_2_Se_6_ as a Promising Photocatalyst

Nanosheets were prepared by finely polishing multilayered AgBiP_2_Se_6_ into flakes. A 150 W halogen lamp (THL) was used as the light source to achieve a power density of ≈10 mW cm^−^^2^ for illuminating a 10 µm methylene blue (MB) solution. Each 4 mL MB solution contained 50 mg of AgBiP_2_Se_6_ nanosheets, dispersed uniformly. The solution mixture was first magnetically stirred in the dark for 10 min to reach adsorption‐desorption equilibrium, followed by an additional 10 min of dark incubation. Subsequently, the samples were exposed individually to THL illumination at room temperature for a total duration of 120 min.

## Conflict of Interest

The authors declare no conflict of interest.

## Author Contributions

C.H.H. conceived the idea and supervised the optical, electrical, and structural characterizations. C.H.H. and Y.X.X. grew the crystals. Y.H.P. and Y.X.X did HRTEM, EDS, and XPS measurements. A.M.U. performed the structural, optical, and electrical measurements. Y.C.S. and A.M.U. did Kelvin‐probe work function measurement, fabricated the device, and performed the device characterization. A.M.U. did photocatalysis application. C.H.H. and A.M.U. wrote the manuscript and analyzed the calculation and experimental results.

## Supporting information



Supporting Information

## Data Availability

The data that support the findings of this study are available from the corresponding author upon reasonable request.
