# Supplementary material for: Exploration of Quasi‐Direct Band Edge in a Multilayer Ferroelectric Semiconductor for Applications in Van Der Waals Stacked Heterojunction Solar Cell and Photocatalytic Devices
Source: Adv Sci (Weinh). 2025 Oct 8;13(2):e14717. doi: 10.1002/advs.202514717 (PMC12786303; doi:10.1002/advs.202514717)
Supplement: Supplementary file 1 — Supporting Information [file ADVS-13-e14717-s001.pdf]

Supplementary Information

of

Exploration of Quasi-direct Band Edge in a Multilayer Ferroelectric Semiconductor for Applications in van der Waals Stacked Heterojunction Solar Cell and Photocatalytic Devices

Anna Milatul Ummah[a], Yen-Chang Su[a], Yu-Hung Peng[a], You-Xun Xu[a], Ching-Hwa Ho[a,b,*]

[a] *Graduate Institute of Applied Science and Technology, National Taiwan University of Science and Technology, Taipei 106, Taiwan*

[b] *Taiwan Consortium of Emergent Crystalline Materials (TCECM), National Science and Technology Council, Taipei, 106, Taiwan*

[*]E-mail: chho@mail.ntust.edu.tw

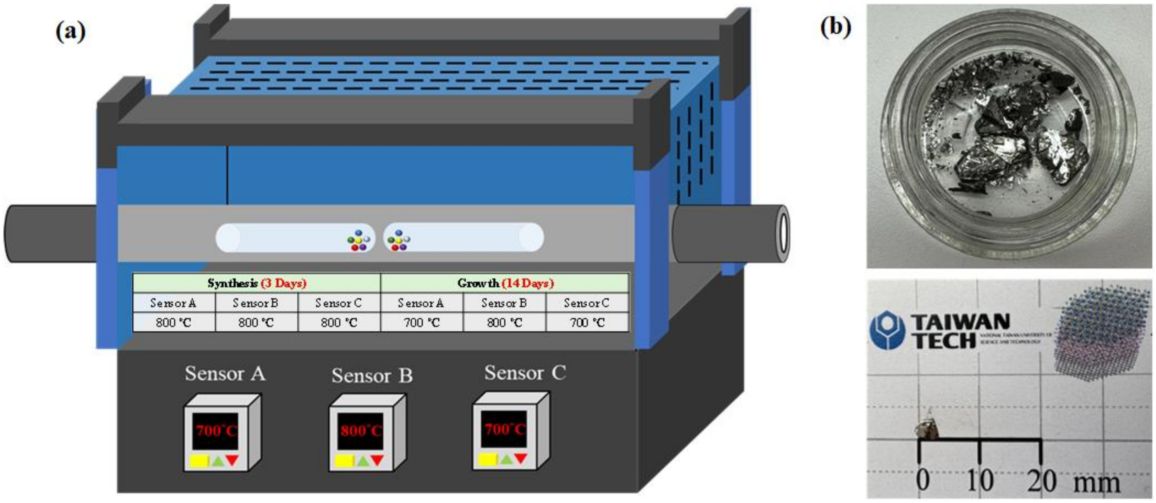

Figure S1. The growth process of $AgBiP_2Se_6$ using CVT method with ICl_3 as the transport agent. (a) The position of crystal tube inside the furnace. The inset table shows the temperature setting for each sensor of the furnace in synthesis and growth condition. (b) The as-grown $AgBiP_2Se_6$ crystals exhibit grey color and sheet-like shiny surface. The bottom image shows the scale size of one bulk $AgBiP_2Se_6$ crystal.

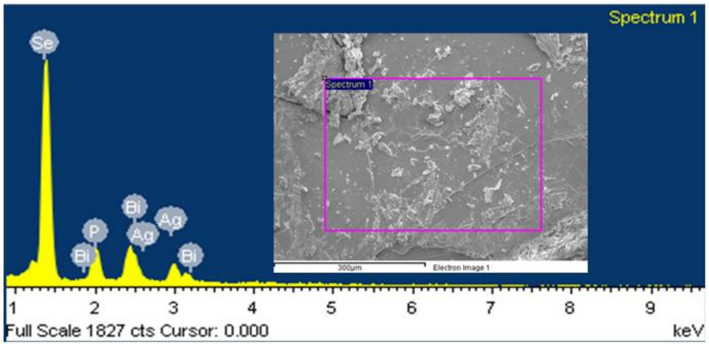

$AgBiP_2Se_6$		
Element	Atomic(%)	Ideal(%)
Ag	9.96	10
Bi	9.91	10
P	21.83	20
Se	58.31	60
Total	100	100

Figure S2. The Energy-Dispersive X-Ray Spectrometry (EDS) of $AgBiP_2Se_6$. The inset shows the scanning electron microscope (SEM) images of ML-$AgBiP_2Se_6$. The table shows the stoichiometric ratio of Ag, Bi, P, and Se element are well matched with the ideal composition in $AgBiP_2Se_6$.

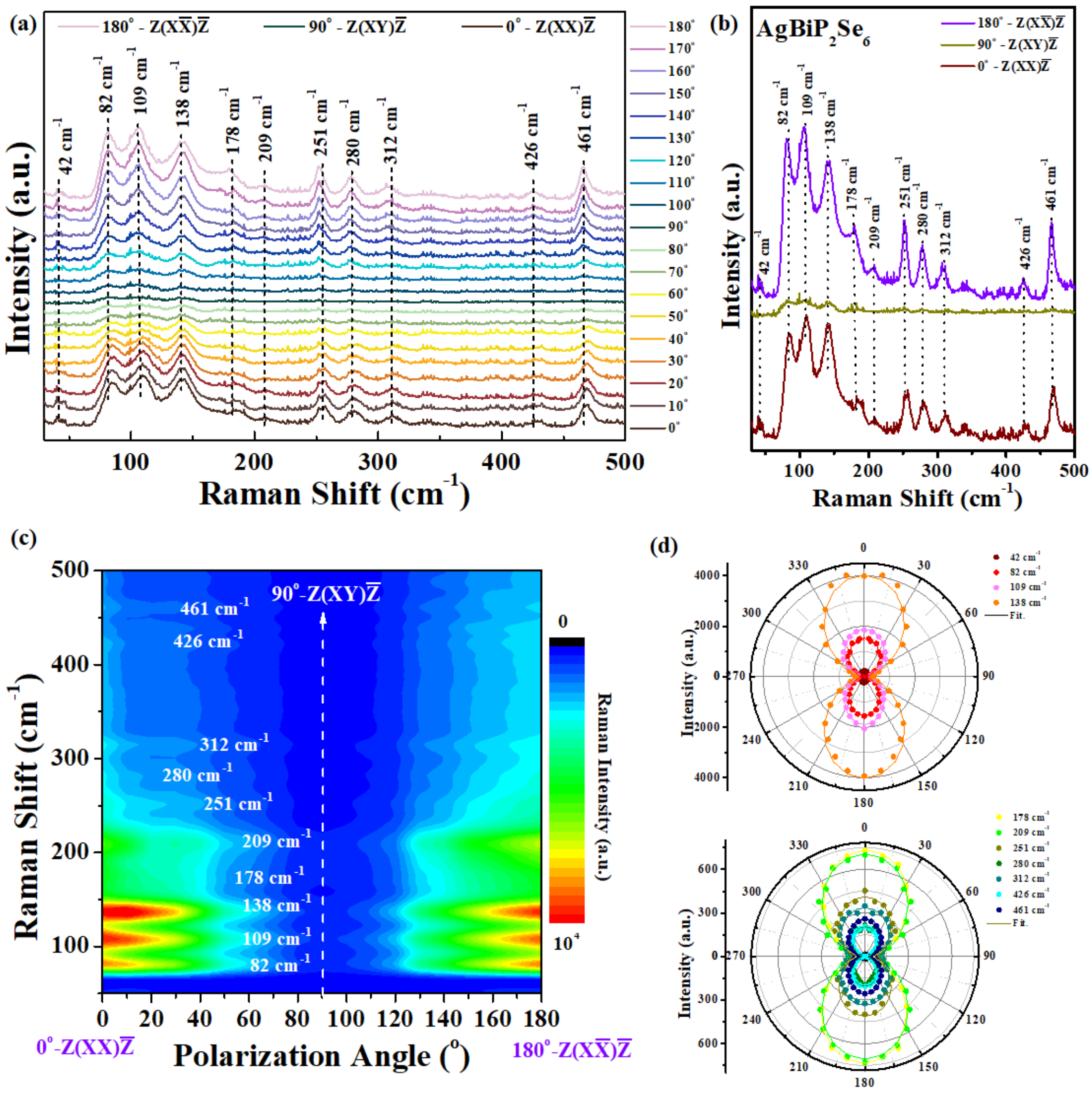

Figure S3. The polarized micro-Raman (μRaman) results of ML-$AgBiP_2Se_6$ at RT. (a) The angle-dependent μRaman spectra in $AgBiP_2Se_6$ from 0° to 180° with an interval of 10° observed in the wavenumber range of 30-500 cm^{-1}. (b) The magnification of polarized μRaman spectra at 0°, 90° and 180°. The spectra show an anisotropy behavior with the peak intensity disappeared at 90°. However, the Raman modes of 82 cm^{-1}, 109 cm^{-1} and 138 cm^{-1} still show small intensity at 90° with the high Raman intensity changing compared to 0° and 180°. (c) The 2D contour plot in polarized μRaman spectra of ML-$AgBiP_2Se_6$ for each Raman mode. (d) The polar plot of polarized μRaman in ML-$AgBiP_2Se_6$ at RT observed below 150 cm^{-1} (top) and between 151-500 cm^{-1} (bottom).

Table S1. The polar plot fitting parameter and polarized rejection ratio [$R=(I_P-I_0)/I_P$, i.e. maximum rejection at R=1] obtained from polarized μRaman analyses of $AgBiP_2Se_6$ at RT.

**Raman shift of $AgBiP_2Se_6$**	$I_\theta = I_0 + I_P \cdot cos^2\ (\theta - \theta_m)$			
	**I_0**	**I_P**	θ_m	**R: $(I_P - I_0)/I_P$**
**42 cm^{-1}**	28.003 ± 0.3	199.676 ± 2	1.094 ± 2	0.86
**82 cm^{-1}**	86.975 ± 0.5	879.028 ± 5	1.062 ± 2	0.901
**109 cm^{-1}**	108.706 ± 0.5	1029.296 ± 7	1.078 ± 2	0.894
**138 cm^{-1}**	92.976 ± 1	946.216 ± 5	2.118 ± 3	0.902
**178 cm^{-1}**	30.201 ± 0.4	332.267 ± 2	2.175 ± 3	0.909
**209 cm^{-1}**	25.437 ± 0.8	191.596 ± 4	1.096 ± 2	0.867
**251 cm^{-1}**	26.999 ± 0.5	442.382 ± 2	1.032 ± 2	0.939
**280 cm^{-1}**	22.898 ± 0.7	317.281 ± 2	2.105 ± 3	0.928
**312 cm^{-1}**	29.801 ± 0.2	244.584 ± 2	1.062 ± 2	0.878
**426 cm^{-1}**	20.186 ± 0.2	140.237 ± 5	2.113 ± 3	0.856
**461 cm^{-1}**	26.473 ± 0.5	488.525 ± 4	1.058 ± 2	0.946

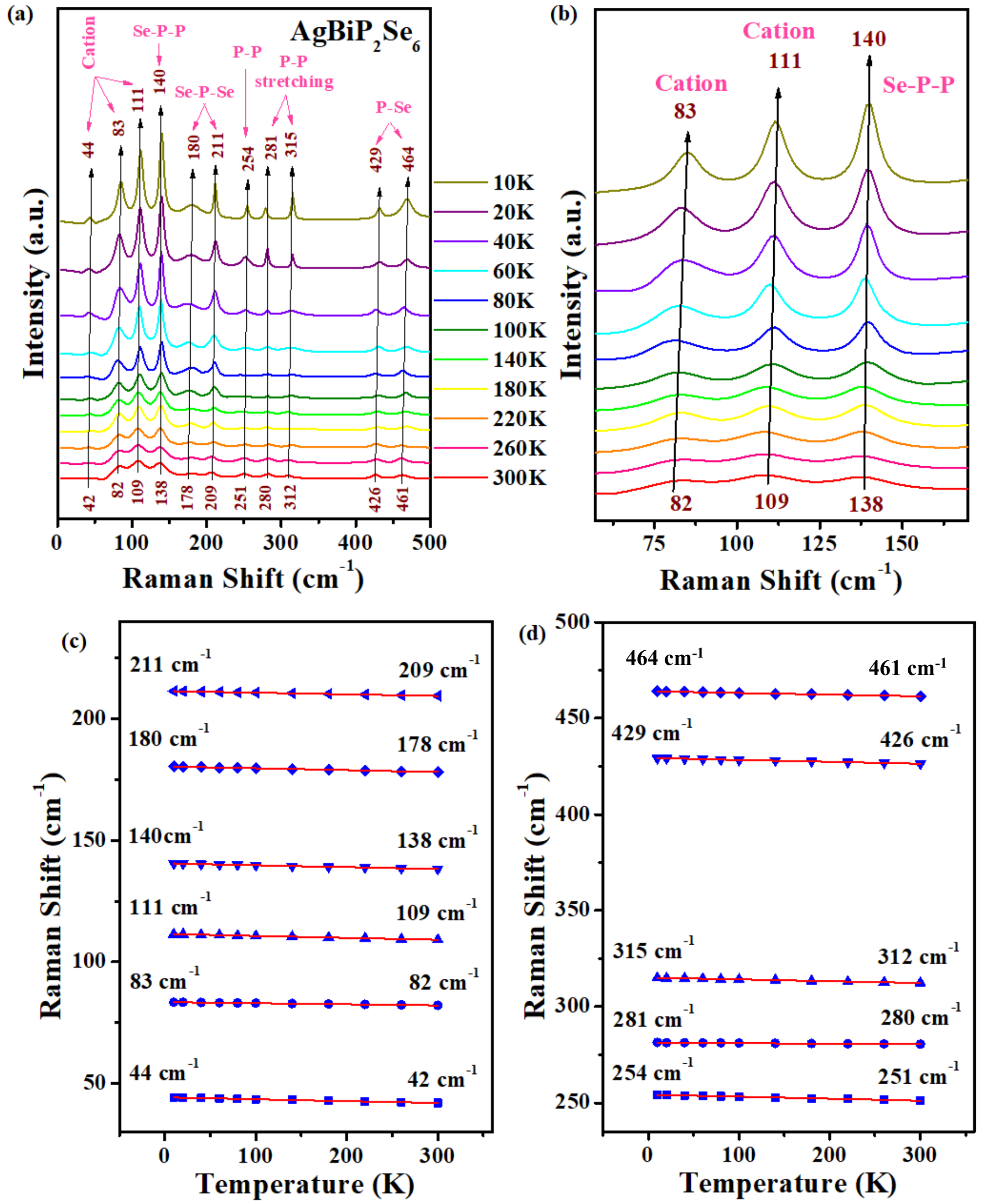

Figure S4. Temperature dependent micro-Raman (μRaman) results of ML-$AgBiP_2Se_6$. (a) The μRaman spectra of $AgBiP_2Se_6$ observed in the Raman shift range of 10-500 cm^{-1} from 300 K down to 10 K. (b) The enlargement of Raman spectra for the three highest intensity peaks in ML-$AgBiP_2Se_6$. (c) The linear fitting of Raman mode below 250 cm^{-1} and (d) above 250 cm^{-1} from 10 to 300 K. The blue symbols represent the experimental data and the red lines indicate the linear fitting result.

Table S2. The linear fitting parameter for the temperature-dependent μRaman spectra at 10-300 K in ML-$AgBiP_2Se_6$ using $\nu_i(T) = \nu_i(0) - m_i.T$ (cm^{-1}) in Fig. S4c and S4d. The attribution of each Raman mode is also included.

ν_i (300) (cm^{-1})	ν_i (10) (cm^{-1})	ν_i (0) (cm^{-1})	m_i ($cm^{-1}K^{-1}$)	Mode	Attribution
42	44	44.388	7.76×10^{-3}	A_g	Ag^+ with $(P_2Se_6)^{4-}$ interaction
82	83	83.509	4.36×10^{-3}	A_g	Bi^{3+} with $(P_2Se_6)^{4-}$ interaction
109	111	111.57	8.095×10^{-3}	E_g	Ag^+ and Bi^{3+} cation interaction
138	140	140.57	7.79×10^{-3}	E_g	Se-P-P bonds
178	180	180.42	7.67×10^{-3}	A_g	Se-P-Se bonds
209	211	211.4	6.65×10^{-3}	A_g	Se-P-Se bonds
251	254	254.27	1.03×10^{-2}	A_g	P-P bonding
280	281	281.39	2.99×10^{-3}	E_g	P-P stretching
312	315	315.11	9.06×10^{-3}	A_g	P-P stretching
426	429	429.37	9.94×10^{-3}	A_g	P-Se oscillation in PSe_3
461	464	464.1	8.82×10^{-3}	A_g	P-Se oscillation in PSe_3

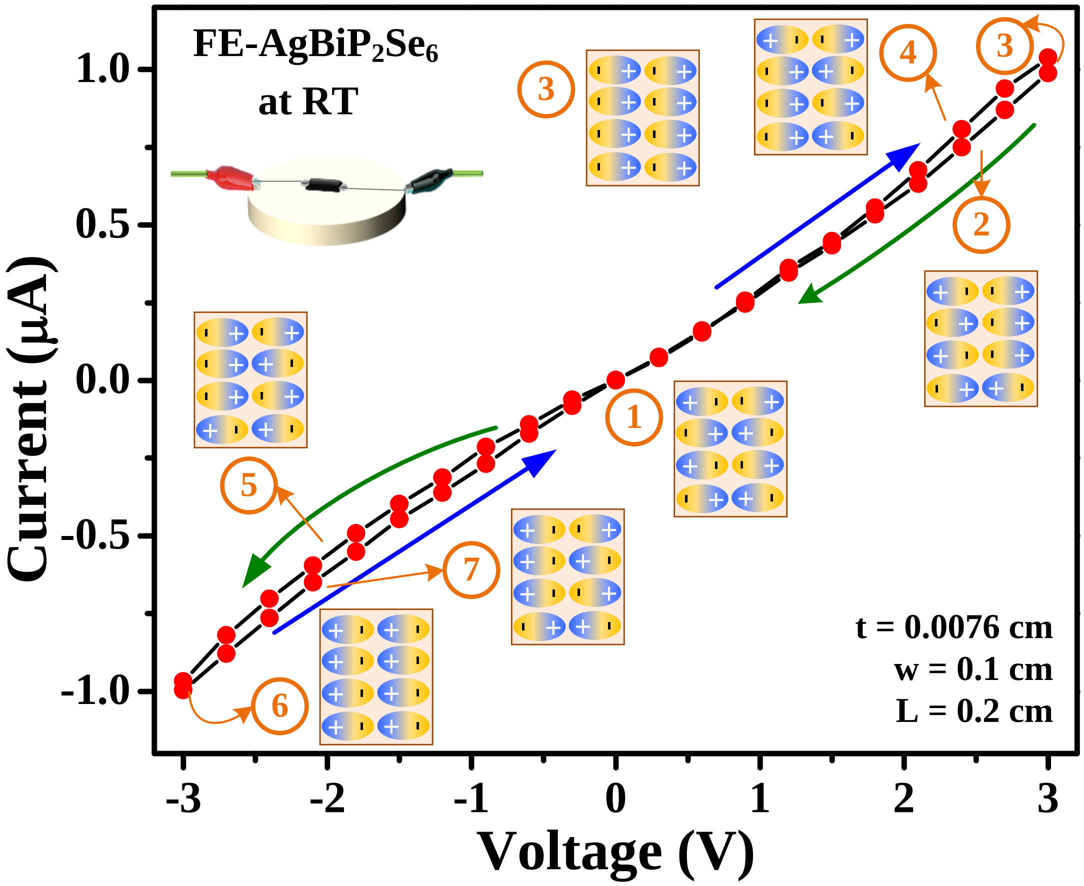

Figure S5. The ferroelectricity investigation of as-grown ML-$AgBiP_2Se_6$ at RT using bidirectional I-V measurements. The I-V curves of ML-$AgBiP_2Se_6$ in the dark condition are measured using two-loop direction measurements: from -3 to 3 V (directed by blue arrow) and from 3 to -3 V (directed by green arrow). The result shows distinct current curves, indicating self-polarization inside the material. The I-V curve shows hysteresis due to the polarization-state switching of ML-$AgBiP_2Se_6$ from positive to negative or from negative to positive voltage for the dipoles in the FE material. The hysteresis curve presents 7 states based on the dipoles' variation in the FE-$AgBiP_2Se_6$. State 3 (applied +3 V) shows the polarization (P) fully aligned in one direction (with "+" toward the right electrode). State 6 (applied -3V) shows the polarization fully aligned in the opposite direction.

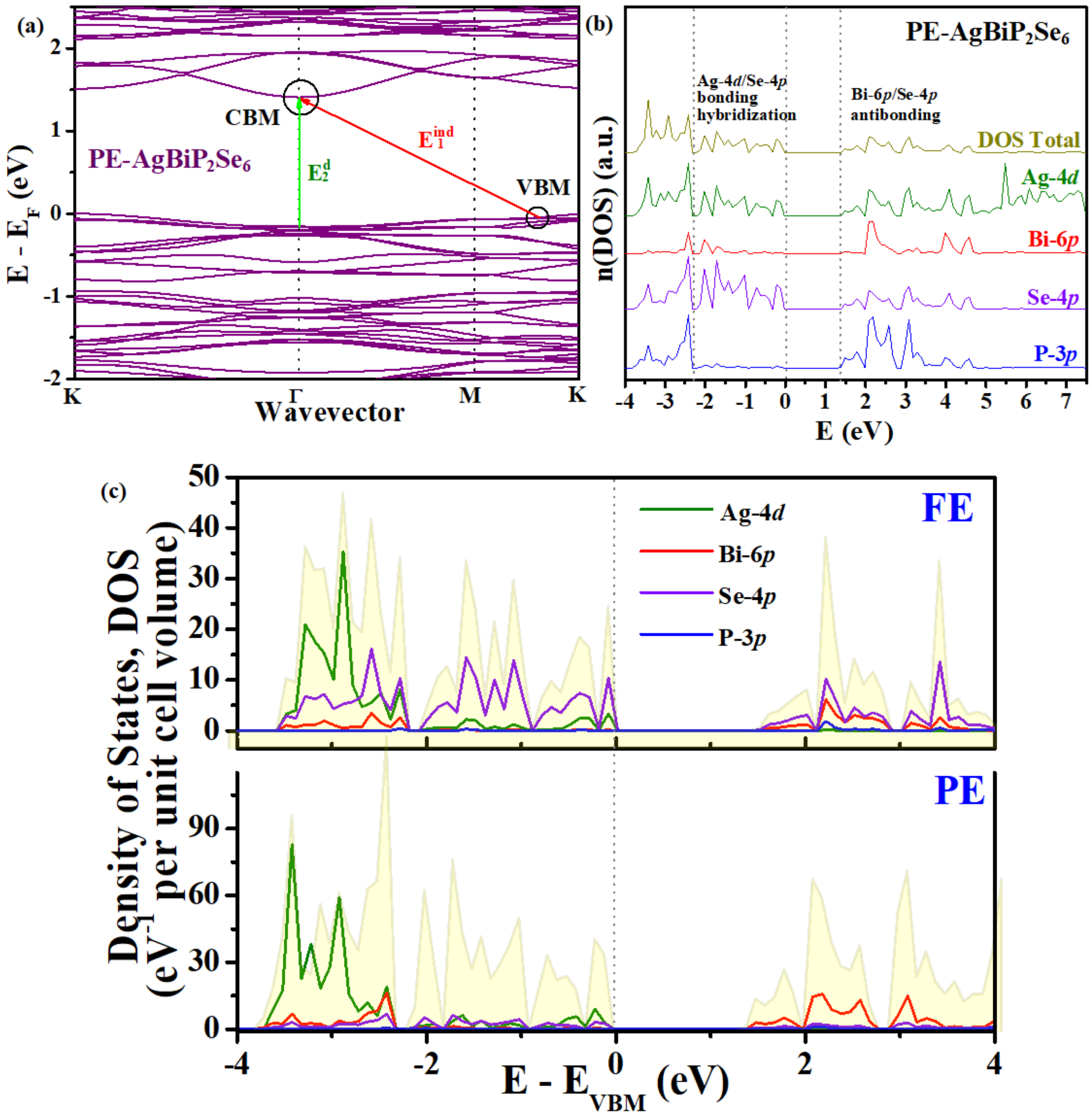

Figure S6. Band structure and density of states (DOS) of paraelectric (PE) single-layer $AgBiP_2Se_6$ calculated using Quantum ESPRESSO simulation package. (a) Electronic band structure of PE $AgBiP_2Se_6$. (b) Normalized density of states in PE $AgBiP_2Se_6$, including the partial DOS (PDOS) in Ag-4*d*, Bi-6*p*, Se-4*p*, P-3*p* orbitals and the total DOS. (c) The DOS comparison of each states in eV^{-1} per unit cell volume in ferroelectric FE (top) and paraelectric PE (bottom) $AgBiP_2Se_6$ calculated using Quantum Espresso.

Table S3. The bandgaps analyzed in FE and PE-phase $AgBiP_2Se_6$.

**Method**	**Thickness**	**Phase**	**Indirect gap (E^{ind})**	**Direct gap (E^{d})**	**Ref.**
Experimental	Bulk	FE	1.4 eV	Not mentioned	S1.
PBE calculation	Monolayer	FE	1.47 eV	Not mentioned	S2.
PBE + U calculation	Bulk	FE	1.384 eV	Not mentioned	S3.
VASP calculation	Monolayer	PE	1.44 eV	Not mentioned	S4.
		FE	1.45 eV	Not mentioned	
Quantum Espresso calculation	Monolayer	PE	1.434 eV	1.496 eV	**This work**
		FE	1.483 eV	1.57 eV	**This work**
Experimental	Multilayer (bulk)	FE	1.46 eV	1.535 eV	**This work**

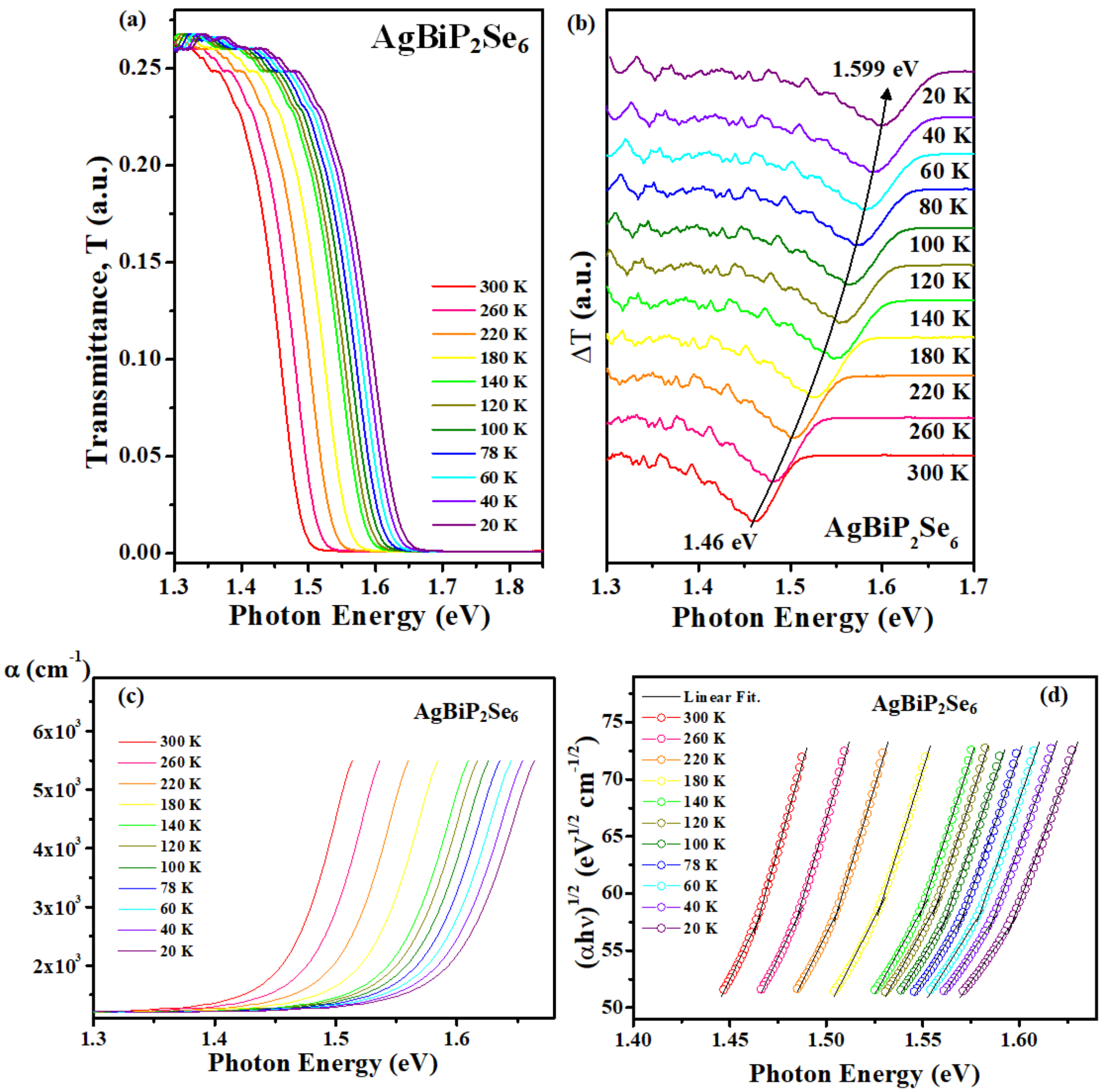

Figure S7. The optical properties of indirect $AgBiP_2Se_6$ through transmittance and absorption characterization. (a) The temperature-dependent transmittance spectra of ML-$AgBiP_2Se_6$ from 300 K to 20 K. (b) The first derivative transmittance spectra of absorption edge of $AgBiP_2Se_6$ derived from (a). (c) The temperature-dependent absorption coefficient obtained by transmittance spectra in (a). (d) The $(\alpha h\nu)^{½}$ vs. hν plot of ML-$AgBiP_2Se_6$ at 20-300 K to observe indirect bandgap and phonon energy using Equ. (2)-(4). The solid black lines indicate the linear fitting result. The curve consists two linear lines: $A_{\alpha_{\mathrm{photon,\ phonon}}}$ corresponds to photon and phonon absorption at lower-energy part and $B_{\alpha_{\mathrm{photon}}+e_{\mathrm{phonon}}}$ corresponds to photon absorption and phonon emission at the higher-energy portion.

Table S4. The obtained fitting parameters of the $(\alpha h\nu)^{1/2}$ vs. hν spectral analysis in the temperature-dependent optical absorption measurements as displayed in Fig. S7d using Equ. (2)-(4).

**Temperature (K)**	$\mathbf{A}_{\alpha_{\mathbf{photon,\ phonon}}}$	$\mathbf{B}_{\alpha_{\mathbf{photon}}+e_{\mathbf{phonon}}}$	**E_g (eV)**	**E_p (eV)**
	**C_1**	**C_2**		
**300**	240.740	464.260	1.46	0.053
**260**	253.880	469.230	1.483	0.048
**220**	269.820	486.530	1.511	0.044
**180**	272.149	483.730	1.53	0.042
**140**	278.460	481.075	1.552	0.05
**120**	303.730	519.150	1.564	0.046
**100**	304.300	522.950	1.566	0.047
**78**	328.980	542.450	1.574	0.044
**60**	340.420	562.480	1.59	0.051
**40**	352.700	567.300	1.595	0.049
**20**	352.080	595.000	1.599	0.048

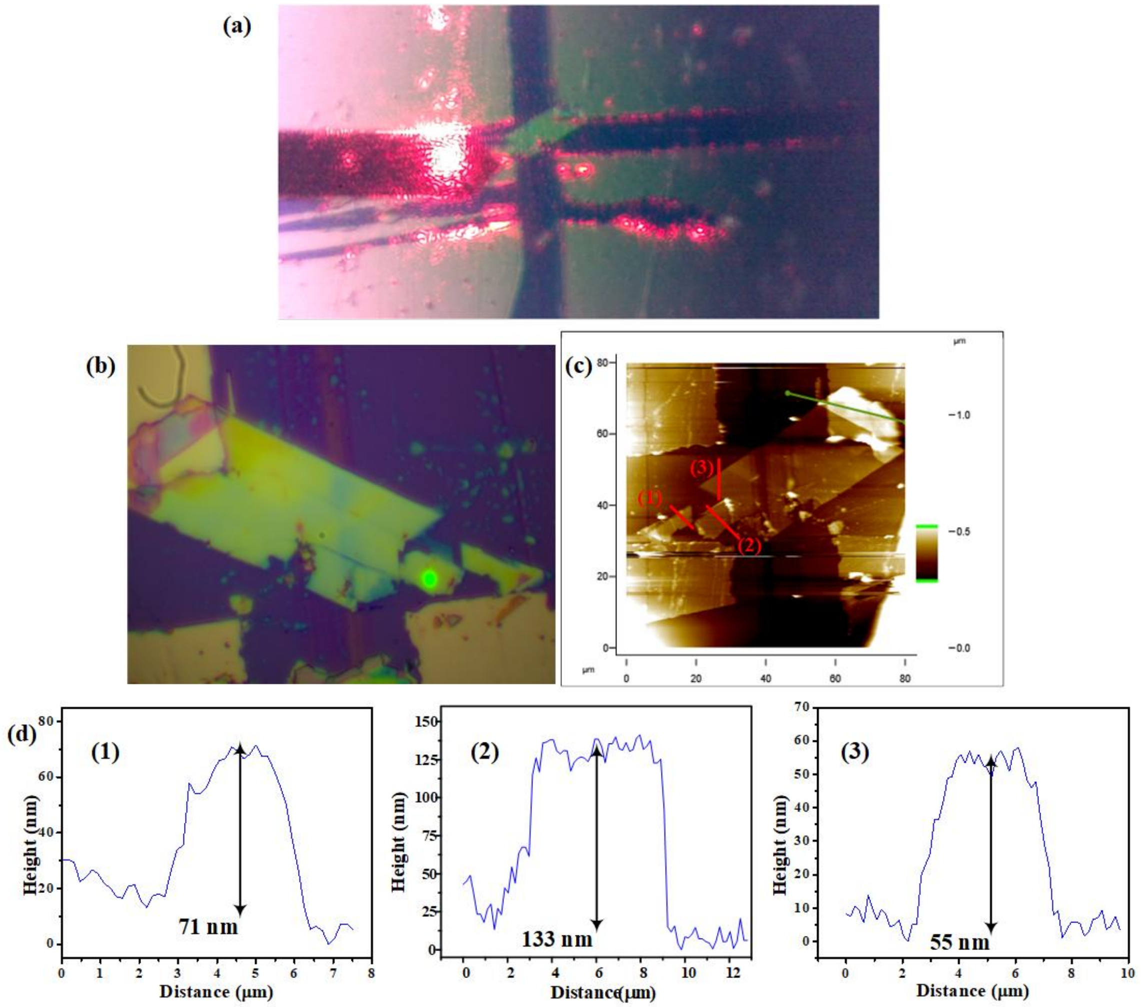

Figure S8. The thickness-measurement process of ML-$AgBiP_2Se_6$ using AFM available for μPL experiment. (a) The optical image of ML-$AgBiP_2Se_6$ during the operation of AFM probing scan process. (b) The OM image of the ML-$AgBiP_2Se_6$ sample consisted of three parts of thickness that are used for thickness-dependent μPL measurement in Fig. 2d. The objective lens of microscope is 50× magnification and a green laser of 532 nm acts as the excitation source. (c) The AFM image of the $AgBiP_2Se_6$ sample consisted of three thickness parts. (d) The thickness-scan profiles of the (1), (2) and (3) parts in the ML-$AgBiP_2Se_6$ as the scanned red lines in (c).

Table S5. The Varshni fitting parameter for micro-thermoreflectance (μTR) and micro-photoluminescence (μPL) spectra in ML-$AgBiP_2Se_6$ using $E_i(T) = E_i(0) - \frac{\alpha_i T^2}{(\beta_i + T)}$.

**Experimental Method**	**Exciton Feature**	**E_i (0) (eV)**	**α_i ($meV.K^{-1}$)**	**β_i (K)**
**μTR**	E_1^{ind}	1.601 ± 0.002	0.841 ± 0.2	250 ± 20
	E_2^{d}	1.674 ± 0.002	0.873 ± 0.08	250 ± 20
	E_3	1.773 ± 0.001	0.811 ± 0.15	250 ± 20
**μPL**	FX (E^d)	1.672 ± 0.01	0.87 ± 0.2	250 ± 20

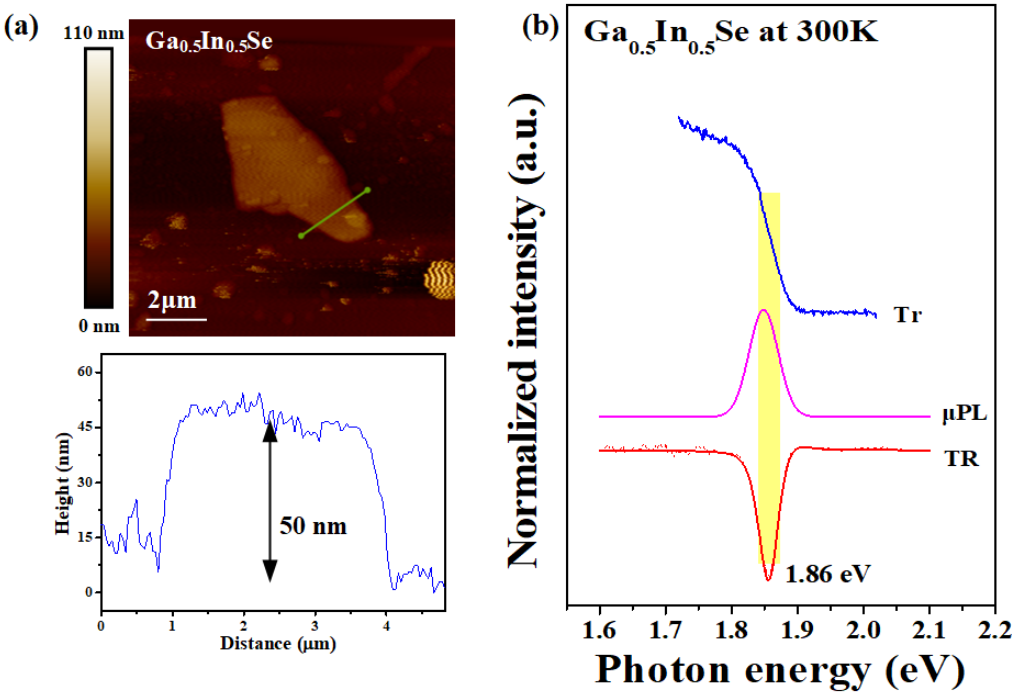

Figure S9. The characterization of optical properties of multilayered $Ga_{0.5}In_{0.5}Se$. (a) The AFM image of ML-$Ga_{0.5}In_{0.5}Se$ (top) to show the thickness of ~50 nm (bottom). (b) The observation of direct gap of ML-$Ga_{0.5}In_{0.5}Se$ measured from optical measurements of transmittance (Tr), µPL, and µTR experiments. The obtained values of direct bandgap of ML-$Ga_{0.5}In_{0.5}Se$ from all optical experiments are matched to be ~1.86 eV (marked with highlight yellow) at 300 K.

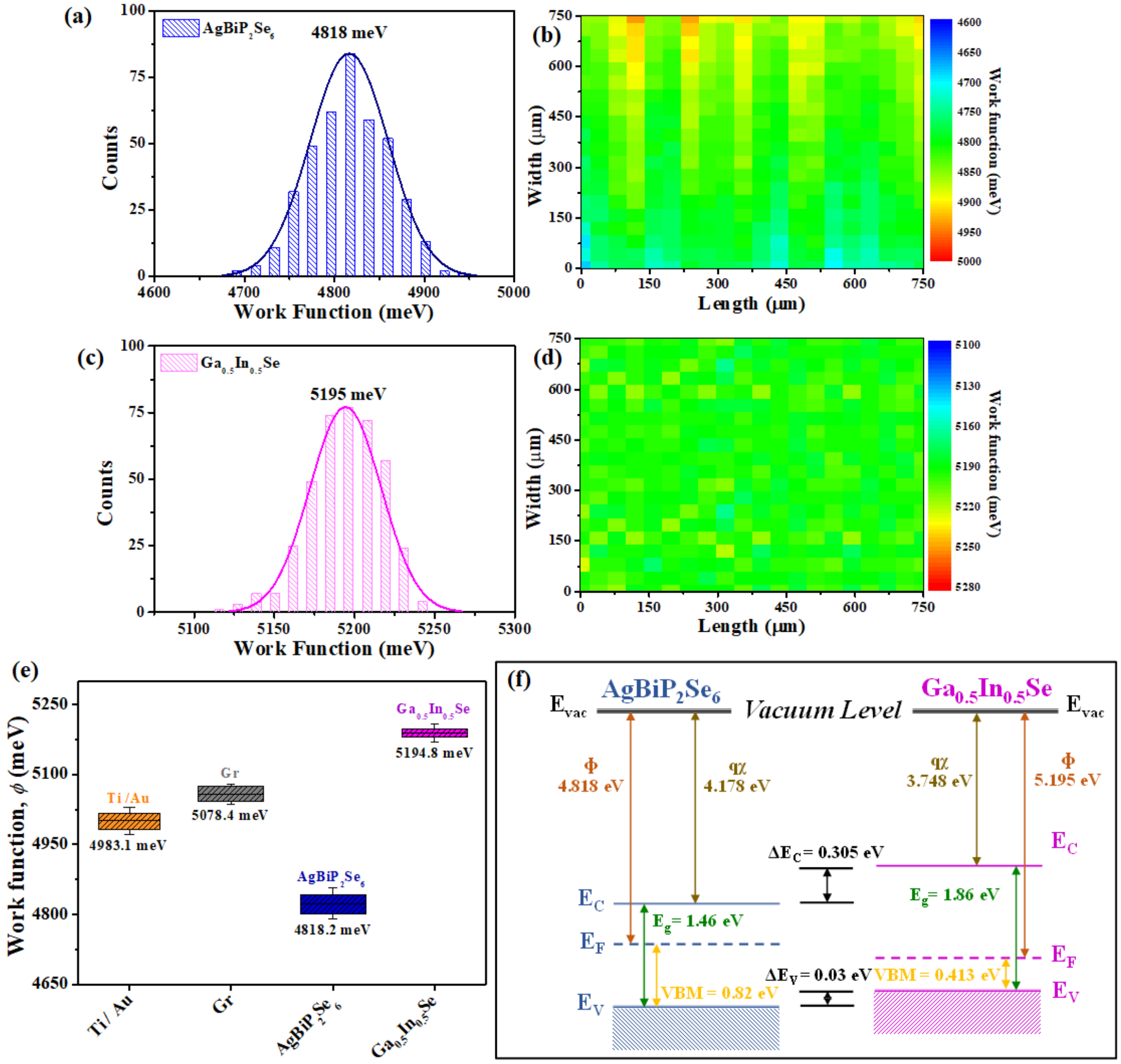

Figure S10. The work function results obtained from Kelvin Probe measurement. These work functions will be used for determining the band diagram of the p-n heterojunction stacking in the $AgBiP_2Se_6/Ga_{0.5}In_{0.5}Se$ heterostructure. (a) The work-function histogram and (b) contour plot in a 750 x 750 μm^2 area of $AgBiP_2Se_6$. (c) The work-function histogram and (d) contour-plot mapping in a 750 x 750 μm^2 area of $Ga_{0.5}In_{0.5}Se$. (e) Work-function comparison of titanium (Ti)/gold (Au), graphene (Gr), $AgBiP_2Se_6$ and $Ga_{0.5}In_{0.5}Se$. (f) The band diagram of separate $AgBiP_2Se_6$ and $Ga_{0.5}In_{0.5}Se$ under thermal equilibrium to determine the band scheme of the stacked heterojunction in Fig 4(b).

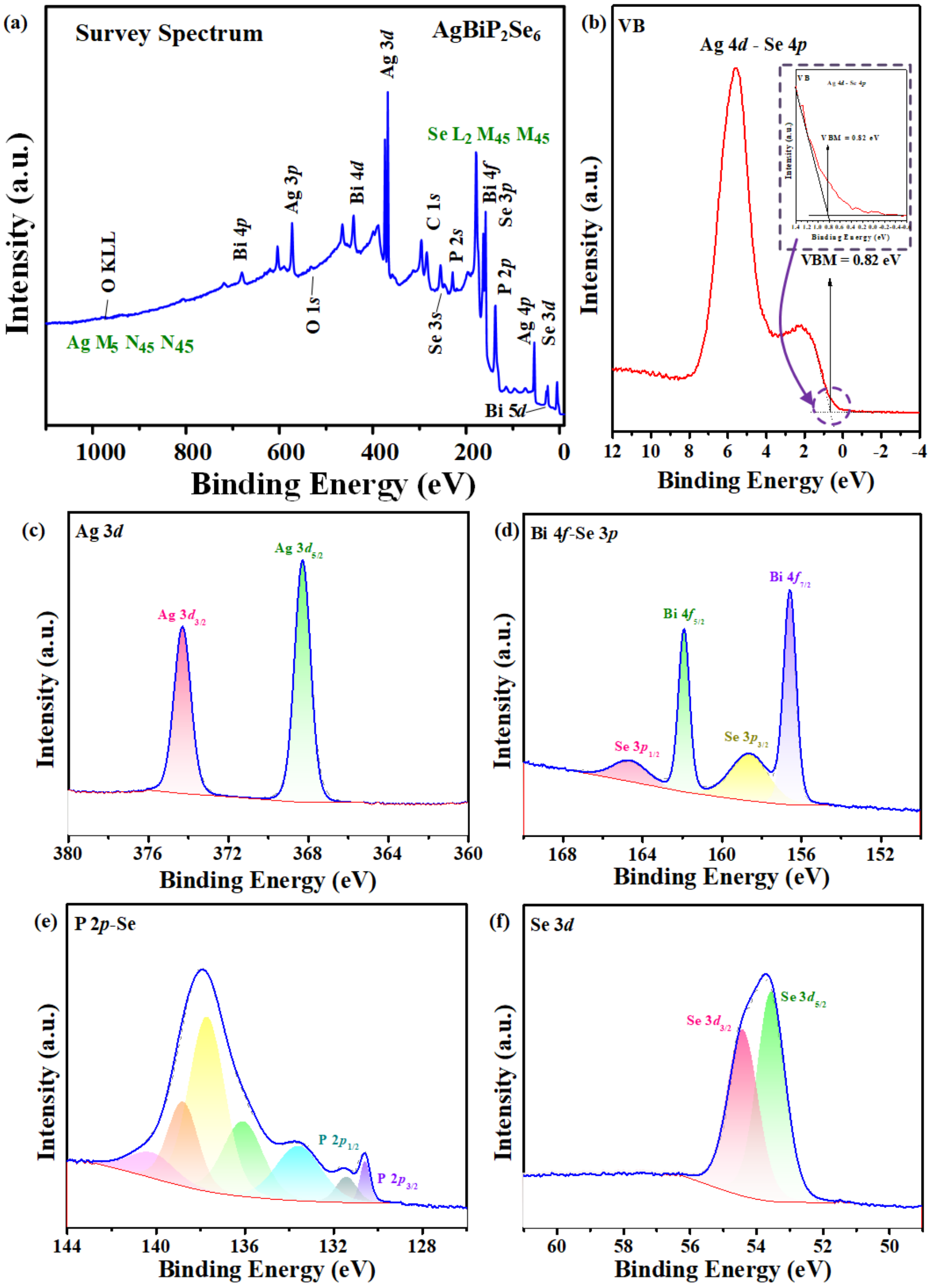

Figure S11. The binding-energy results for $AgBiP_2Se_6$ in the high-resolution XPS spectra. (a) Full XPS spectrum of the $AgBiP_2Se_6$ flake. (b) The valence-band XPS spectrum including linear-extrapolation analysis of binding energy in the range of 12 to -4 eV. The inset shows the analysis of E_F-VBM of ~0.82 eV. The binding energy spectra observed in (c) Ag 3*d*, (d) Bi 4*f*-Se 3*p*, (e) P 2*p*-Se, and (f) Se 3*d*, for $AgBiP_2Se_6$ to identify the crystallinity.

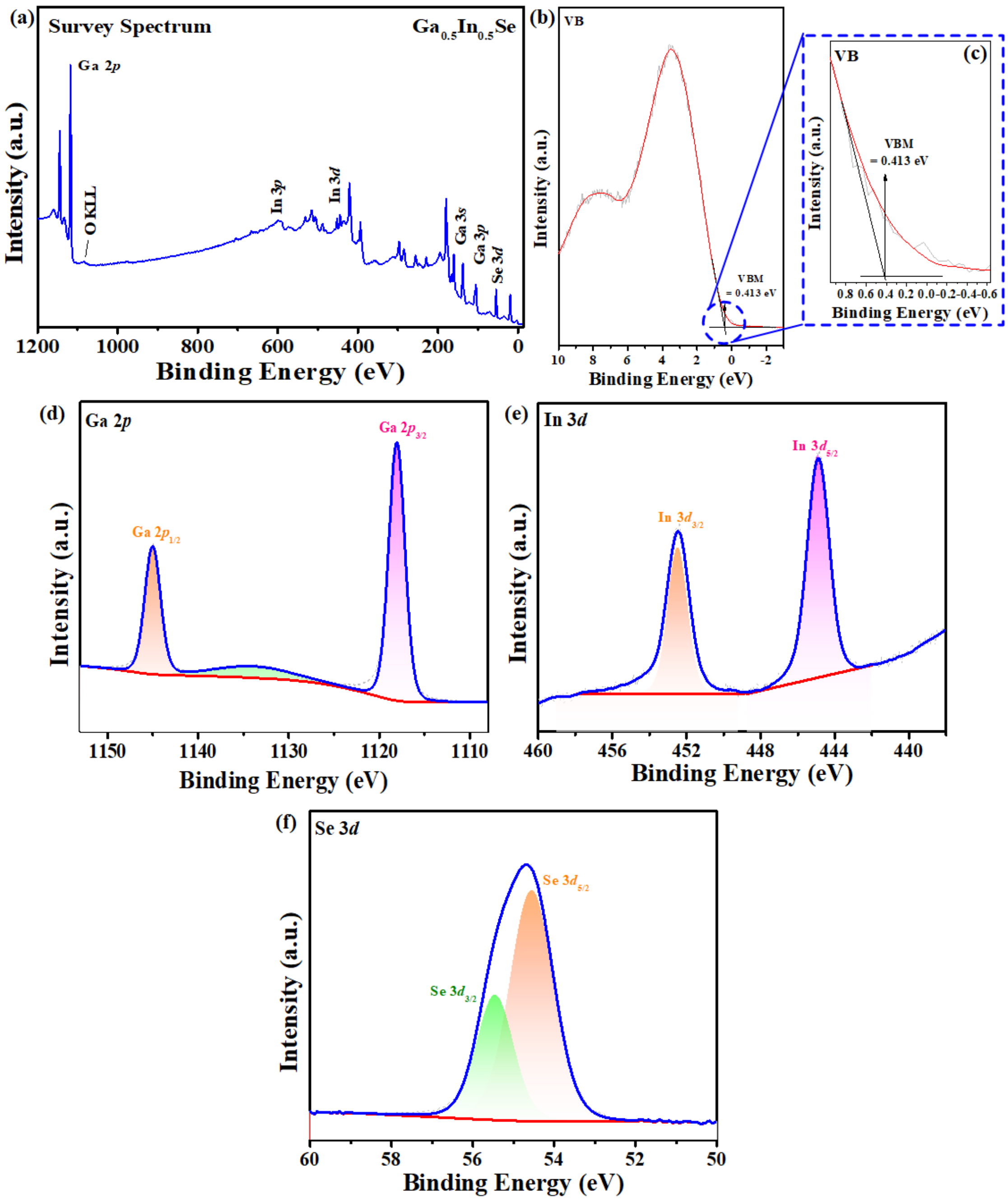

Figure S12. The binding-energy results for $Ga_{0.5}In_{0.5}Se$ in the high-resolution XPS spectra. (a) Full XPS spectrum of $Ga_{0.5}In_{0.5}Se$ layered flake. (b) The VB XPS spectrum of 10 to -2 eV including (c) linear-extrapolation analysis of binding energy. The result indicates the binding energy of E_F-VBM is about 0.413 eV. The binding-energy spectra observed in (d) Ga 2*p*, (e) In 3*d*, and (f) Se 3*d* for $Ga_{0.5}In_{0.5}Se$ to identify its crystal quality.

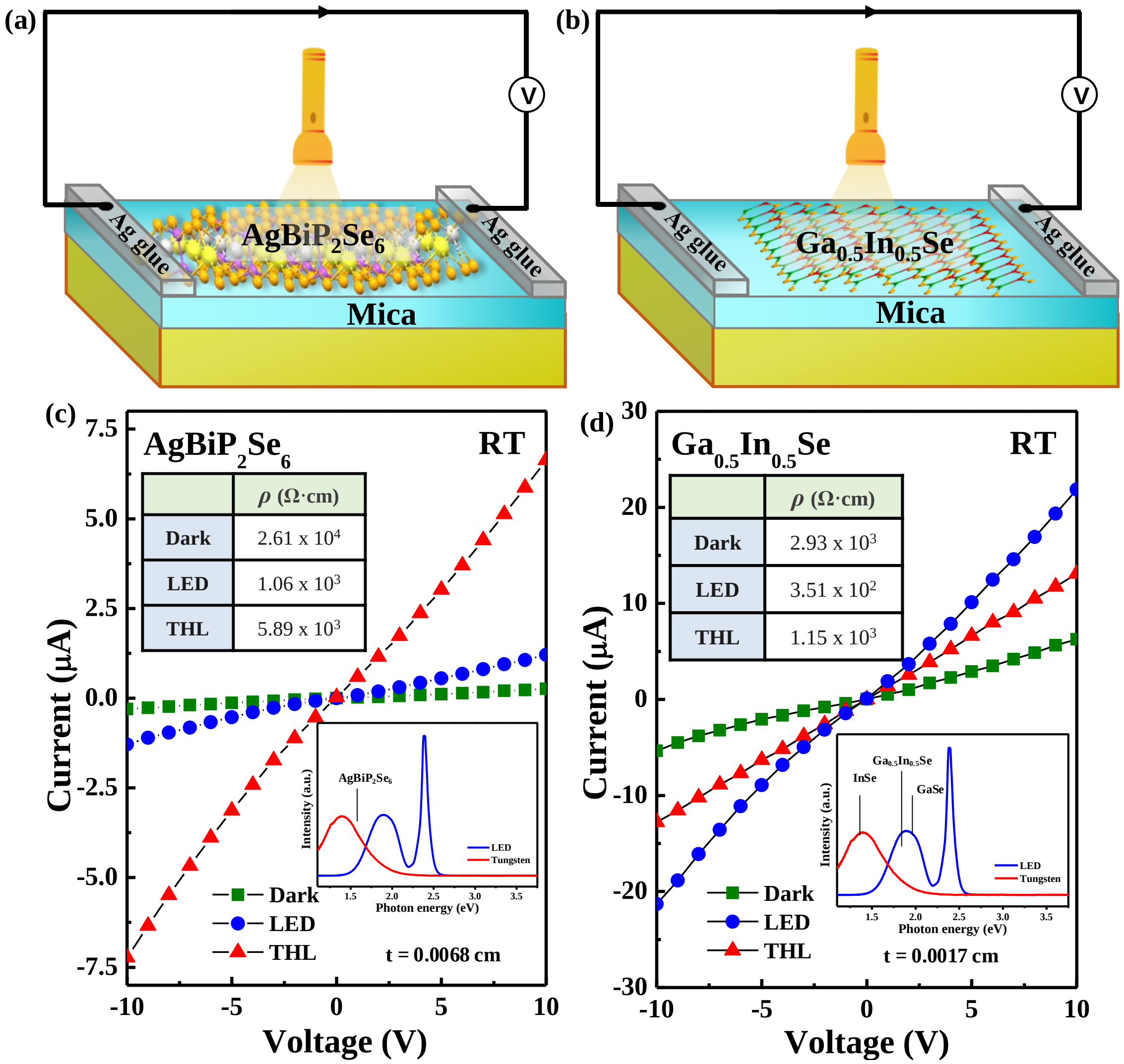

Figure S13. The electrical properties of $AgBiP_2Se_6$ and $Ga_{0.5}In_{0.5}Se$ obtained by I-V measurement. (a) The schematic of sample preparation and operation using layered $AgBiP_2Se_6$ and (b) $Ga_{0.5}In_{0.5}Se$ for Photo I-V measurement with Ag glue as the contact. (c) The Photo I-V plot in the dark condition (pointed by green rectangle dot), under LED (blue circle dot), and under tungsten halogen lamp THL (red triangle dot) illuminations for the bulk $AgBiP_2Se_6$ and (d) for the bulk $Ga_{0.5}In_{0.5}Se$. The inset in figure (c) and (d) shows the LED and THL emission spectra in photon-energy range of 1-4 eV with the bandgap positions of the layer materials are included. The inset table in figure (c) and (d) shows the resistivity results obtained from the corresponding Photo I-V measurement.

Table S6. Electrical and carrier transport properties of $AgBiP_2Se_6$ and $Ga_{0.5}In_{0.5}Se$ measured using resistivity, hot probe and Hall measurements at RT.

Material	Resistivity at RT (Ω·cm)	Carrier concentration (cm^{-3})	Hall mobility ($cm^2/V·Sec$)	Hall carrier type	S (V/K)	Band gap (eV)
$AgBiP_2Se_6$	1.15×10^3	4.16×10^{13}	131	n-type	-1.03×10^{-2}	1.46
$Ga_{0.5}In_{0.5}Se$	7.62×10^2	3.36×10^{13}	244	p-type	2.26×10^{-3}	1.86

Table S7. The electrical properties of $AgBiP_2Se_6$ and $Ga_{0.5}In_{0.5}Se$ from Photo I-V measurements in Fig. S13.

Sample	Characteristic	Dark	LED	Tungsten halogen lamp (THL)
$AgBiP_2Se_6$	Resistance, R (Ω)	3.55×10^7	8.01×10^6	1.44×10^6
	Resistivity, ρ (Ω-cm)	2.61×10^4	5.89×10^3	1.06×10^3
$Ga_{0.5}In_{0.5}Se$	Resistance, R (Ω)	1.83×10^6	2.19×10^5	7.21×10^5
	Resistivity, ρ (Ω-cm)	2.93×10^3	3.51×10^2	1.15×10^2

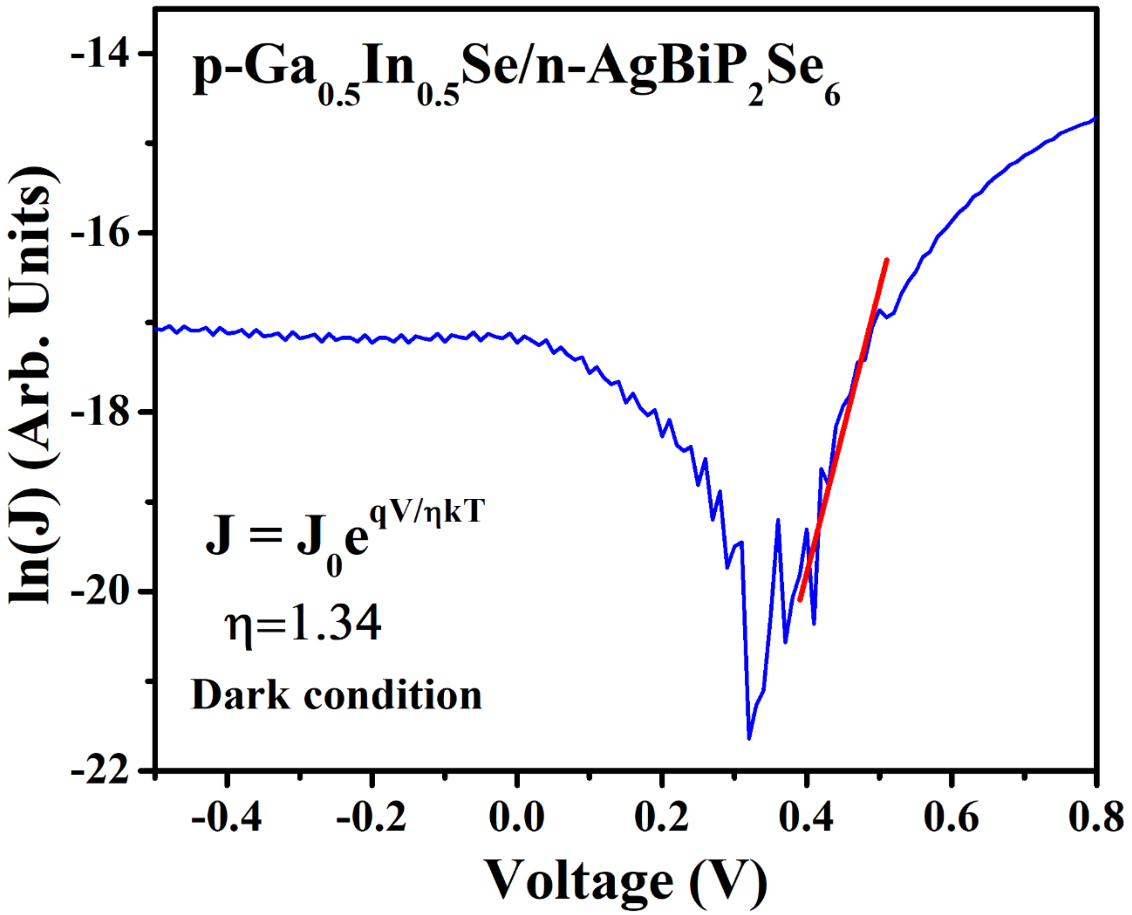

Figure S14. The logarithmic of current density (J) versus voltage (V) curve to analyze the diode ideality factor (η) of the p-$Ga_{0.5}In_{0.5}Se$/n-$AgBiP_2Se_6$ stacked heterojunction derived from Fig. 4h under dark condition. The curve exhibits an ideality factor η of ~1.34 for the heterojunction diode.

Table S8. The comparison of the photocatalyst performance in 2D materials using methylene blue (MB) as the pollutant dye.

Sample	Sample dose (mg)	MB dye concentration	Light source	Irradiation time (min)	Degradation efficiency (%)	Degradation rate, k	Ref.
$AgBiP_2Se_6$	50	10 µM	THL	120	82.4	2.52 x 10^{-3} min^{-1}	**This work**
VS_2	5	50 mL dilute MB solution (5 mg/500 mL of DI water)	5W of LED	105	99	1.8 x 10^{-2} min^{-1}	S5.
SnS_2	5		5W of LED	105	15	7.4 x 10^{-4} min^{-1}	
WS_2	5		5W of LED	105	15	8.3 x 10^{-4} min^{-1}	
$SnS_{1.2}Se_{0.8}$	20	50 µM	XAL	120	69.5	9.99 x 10^{-3} min^{-1}	S6.
$GaTe_{0.6}S_{0.4}$	0.5g/L	10 mg/L	THL	120	97	~3.37 x 10^{-2} min^{-1}	S7.
ZnS	100	10 mg/L	Halogen Lamp	360	30	1.09 x 10^{-3} min^{-1}	S8.
CdS	100	10 mg/L		360	63	2.98 x 10^{-3} min^{-1}	
$MnTiO_3$	5mg/ 50mL	1 x 10^{-5} M	Sunlight	240	62.5	5.25 x 10^{-3} min^{-1}	S9.

References

S1. M. A. Gave, D. Bilc, S. D. Mahanti, J. D. Breshears, M. G. Kanatzidis, On the Lamellar Compounds $CuBiP_2Se_6$, $AgBiP_2Se_6$ and $AgBiP_2S_6$. Antiferroelectric Phase Transitions Due to Cooperative Cu^+ and Bi^{3+} Ion Motion. *Inorg. Chem.***44**, 5293-5303 (2005).

S2. B. Xu, H. Xiang, Y. Xia, K. Jiang, X. Wan, J. He, J. Yin, Z. Liu, Monolayer $AgBiP_2Se_6$: an atomically thin ferroelectric semiconductor with out-plane polarization. *Nanoscale* **9**, 8427–8434 (2017).

S3. T. V. Vu, O. Y. Khyzhun, A. A. Lavrentyev, B. V. Gabrelian, V. I. Sabov, M. Y. Sabov, M. Y. Filep, A. I. Pogodin, I. E. Barchiy, A. O. Fedorchuk, B. Andriyevsky, M. Piasecki, Highly anisotropic layered crystal $AgBiP_2Se_6$: Growth, electronic band-structure and optical properties. *Mat. Chem. Phys.* **277**, 125556 (2022).

S4. S. Lv, J. Wang, B. Wei, Z. Wang, Synthesis, atomic structure and electronic properties of ferroelectric $AgBiP_2Se_6$ ultrathin flakes. *J. Alloys Compd.* **996**, 174803 (2024).

S5. A. Joseph, P.M. Aneesh. Efficient degradation of methylene blue: A comparative study using hydrothermally synthesised SnS_2, WS_2 and VS_2 nanostructures. *Mater. Res. Bull.* **146**, 111623 (2022).

S6. T. M. Herninda, Z-. Y. Chen, C-. H. Ho. Structural, optical and electrical properties in multilayer $SnS_{2(1-x)}Se_{2(x)}$ compounds for energy, thermoelectric and photocatalytic application. *Mater. Today Adv.* **22**, 100498 (2024).

S7. L. C. Muhimmah, Y-. H. Peng, C-. H. Ho. Light emission, structure-phase evolution, and photocatalytic behavior in full-series multilayered $GaTe_{1-x}S_x$ ($0 \leq x \leq 1$) with direct-transition edge. *Mater. Today Adv.* **21**, 100450 (2024).

S8. N. Soltani, E. Saion, M. Z. Hussein, M. Erfani, A. Abedini, Gh. Bahmanrokh, M. Navasery, P. Vaziri. Visible Light-Induced Degradation of Methylene Blue in the Presence of Photocatalytic ZnS and CdS Nanoparticles. *Int. J. Mol. Sci.* **13**, 12242-12258 (2012).

S9. S. Alkaykh, A. Mbarek, E. E. A-. Shattle. Photocatalytic degradation of methylene blue dye in aqueous solution by $MnTiO_3$ nanoparticles under sunlight irradiation. *Heliyon*. **6**, e03663 (2020).
